# Autoantibodies against glucose-regulated protein 78 as serological biomarkers in metastatic and recurrent hepatocellular carcinoma

**DOI:** 10.18632/oncotarget.15192

**Published:** 2017-02-08

**Authors:** Xia Ying, Su-xia Han, Chen-chen He, Cong-ya Zhou, Yi-ping Dong, Meng-jiao Cai, Xin Sui, Cheng-xian Ma, Xiao Sun, Yuan-yuan Zhang, Wen-li Gou, Clifford Mason, Qing Zhu

**Affiliations:** ^1^ Department of Oncology, The Second Affiliated Hospital of Xi'an Jiaotong University Medical College, Xi’an, Shannxi, P.R. China; ^2^ Department of Gynecological Oncology, Women's Hospital, School of Medicine, Zhejiang University, Hangzhou, Zhejiang, P.R. China; ^3^ Department of Oncology, The First Affiliated Hospital of Xi’an Jiaotong University Medical College, Xi’an, Shannxi, P.R. China; ^4^ Department of Obstetrics and Gynecology, The First Affiliated Hospital of Xi’an Jiaotong University Medical College, Xi’an, Shannxi, P.R. China; ^5^ Department of Obstetrics and Gynecology, University of Kansas School of Medicine, Kansas City, Kansas, USA

**Keywords:** heptocellular carcinoma (HCC), tumor associated antigens (TAA), proteomics, autoantibodies, biomarker

## Abstract

**Purpose:**

To identify Heptocellular carcinoma (HCC) associated antigens by proteomics, and validate whether autoantibodies against tumor-associated antigens (TAAs) could be used for diagnosis and conditional monitoring.

**RESULTS:**

The 78 kDa glucose regulated protein (GRP78) was selected as a candidate TAA. The titers of autoantibodies against 78 kDa glucose regulated protein (GRP78) from patients with HCC, liver cirrhosis (LC), and chronic hepatitis (CH) were significantly higher than that from normal controls (*P*<0.05, *P*<0.001, and *P*<0.01, respectively). The expression of autoantibodies against GRP78 was associated with clinical stage (*P*<0.01), portal vein invasion (*P*<0.05), and metastasis (*P*<0.05). The expression of anti-GRP78 antibodies was significantly higher 1 month after surgery in recurrent patients who had accepted hepatic resection 1 month after surgery compared to patients who had surgery before surgery or within 1 week after surgery (*P*<0.01 and *P*<0.001). Immunohistochemistry (IHC) showed higher expression of GRP78 in HCC compared to the non-HCC liver tissues (*P* <0.05).

**Materials and Methods:**

HCC serum with high titer of autoantibodies against TAAs were screened and used for a proteome-based approach to identify HCC associated antigens. Indirect enzyme-linked immunoassay (ELISA) was used to detect the corresponding autoantibodies against TAAs.

**Conclusion:**

GRP78 is an autoantigen that could stimulate autoimmune responses and serve as a potential marker for recurrent and metastatic progression in HCC.

## INTRODUCTION

Hepatocellular carcinoma (HCC) is one of the leading causes of cancer-related deaths worldwide [1]. The majority of patients with HCC are usually in advanced stages of disease with poor prognosis. Alpha fetal protein (AFP) is a common serum marker of HCC, however, its sensitivity and specificity are not optimal [2, 3]. Therefore, identifying new biomarkers of HCC is of great clinical importance. Studies have demonstrated that antigenic changes in cells can be recognized by the immune system of patients and elicit an immunoreaction [4, 5]. Many autoimmune diseases such as systemic lupus erythematosus (SLE) [6], diabetes [7], and rheumatoid arthritis (RA) [8] have been shown to have autoantibody responses. Many nonautoimmune diseases such as cancer [9–11] also have autoantibody responses-TAAs are cellular proteins whose aberrant regulation of function is linked to malignancy. Autoantibodies stimulated by TAAs are involved in tumorigenesis. Therefore, these autoantibodies could be used as probes to identify antigens that are potentially involved in malignant transformation. Several approaches are available for the identification of TAAs in cancer. For instance, serum antibodies from patients can be used to screen cDNA libraries to identify TAAs with potential value as biomarkers for cancer diagnosis. Several studies have used this approach to identify TAAs such as p62 and p90 [12, 13]. Compared to the cDNA expression library immunoscreen approach, proteomics technology can be used to identify a large number of antigens and distinguish isoforms and detection of autoantibodies directed against post-translational modifications (PTMs) of targets [14, 15]. In this study, HCC serum with high titer of autoantibodies against TAAs were screened and identified. The results were used to determine whether autoantibodies to TAAs could be used for diagnosis and conditional monitoring in HCC.

## RESULTS

### Prevalence of autoantibodies in patients with hepatocellular carcinoma

**Figure 1:**
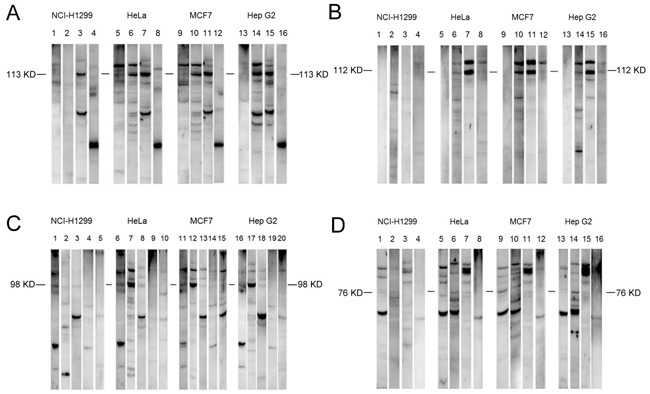
Detection of autoantibodies against cellular proteins in sera from patients with liver diseases **A**. Detection of autoantibodies against 113-kDa cellular proteins in sera from patients with liver diseases. NHS sera 147: lanes 1, 5, 9, and 13; HCC sera 169: lanes 2, 6, 10, and 14; HCC sera 65: lanes 3, 7, 11, and 15; LC sera 14: lanes 4, 8, 12, and 16. **B**. Detection of autoantibodies against 112-kDa cellular proteins in sera from patients with liver diseases. NHS sera 84: lanes 1, 5, 9, and 13; HCC sera 113: lanes 2, 6, 10, and 14; HCC sera 493: lanes 3, 7, 11, and 15; CH sera 54: lanes 4, 8, 12, and 16. **C**. Detection of autoantibodies against 98-kDa cellular proteins in sera from patients with liver diseases. NHS sera 31: lanes 1, 6, 11, and 16; HCC sera 244: lanes 2, 7, 12, and 17; HCC sera 134: lanes 3, 8, 13, and 18; LC sera: lanes 4, 9, 14, and 19; CH sera 14: lanes 5, 10, 15, and 20. **D**. Detection of autoantibodies against 76-kDa cellular proteins in sera from patients with liver diseases. NHS sera 121: lanes 1, 5, 9, and 13; HCC sera 534: lanes 2, 6, 10, and 14; HCC sera 162: lanes 3, 7, 11, and 15; CH sera 94: lanes 4, 8, 12, and 16. HCC, hepatocellular carcinoma; LC, liver cirrhosis; CH, chronic hepatitis; NHS, normal human serum.

100 HCC patients, 30 LC patients, 30 CH patients, and 30 control patients were recruited to screen serum samples from HCC patients with high titers of autoantibodies. Some patients’ sera contained high titer of autoantibodies against specific cellular antigens. HCC sera were identified by Western blot using antibodies against the unknown 113-kDa proteins, 112-kDa proteins, 98-kDa proteins, and 76-kDa proteins. The HCC sera contained a higher frequency of autoantibodies to these antigens than that in LC, CH, and normal human sera (NHS) groups. Representative Western blots are illustrated in Figure 1 and Supplementary Table 1.

### Identification of candidate proteins by proteomic approach

HCC serum with high titer of autoantibodies was selected to identify candidate proteins. Serum samples and HepG_2_ cell extractions were subjected to immunoprecipitation followed by SDS-PAGE and coomassie blue staining. Target gels were analyzed by LC-MS/MS. In total, 566 proteins were identified (Supplementary Table 2). Eight candidate proteins, including Alpha-actinin-4 (ACTN4), ATP-citrate synthase isoform 2 (ACLY), exportin-2 isoform1 (CSE1L), endoplasmin precursor (TRA1), heat shock protein 105 kDa (HSPH1), heat shock 70 kDa protein 4 (HSPA4), 78 kDa glucose regulated protein precursor (GRP78), and heat shock protein 75 kDa (TRAP1) were selected based on bioinformation standards, test reliability, and targeted molecular weight (Table 1). These proteins were primarily located in the cytoplasm and involved in biological processes such as protein transport, localization, programmed cell death, and associated with molecular functions such as stress response, and antigen processing or presentation (Table 2).

**Table 1:** Partial results of proteins identified by mass spectrometry

No.	Accession no.	Identified proteins	Score	Coverage	Unique peptides	MW(kDa)
1	24234756	interleukin enhancer-binding factor 3 isoform c	24.75	6.09%	3	74.6
2	67191208	polyubiquitin-C	41.81	32.85%	2	77
3	7427519	DNA replication licensing factor MCM6	85.89	9.38%	6	92.8
4	21914927	lymphoid-specific helicase	0	1.19%	1	97
5	4557469	AP-2 complex subunit beta isoform b	56.89	6.40%	5	104.5
6	27477041	AP-2 complex subunit alpha-2 isoform 2	23.59	2.88%	2	103.9
7	209969812	KN motif and ankyrin repeat domain- containing protein 2 isoform 2	0	1.41%	1	91.1
8	188497750	hexokinase-1 isoform HKI-td	58.11	7.51%	6	101
9	367460087	myosin-10 isoform 2	54.93	1.06%	1	228.9
10	69354671	ATP-binding cassette sub-family F member 1 isoform a	0	1.30%	1	95.9
11	77404397	staphylococcal nuclease domain-containing protein 1	60.04	6.04%	5	101.9
12	10800138	histone H2B type 1-D	36.32	15.87%	2	13.9
13	58530840	desmoplakin isoform I	57.13	2.61%	8	331.6
14	16579885	60S ribosomal protein L4	25.46	2.58%	1	47.7
15	6631095	DNA replication licensing factor MCM3	63.65	6.81%	5	90.9
16	31541941	heat shock 70 kDa protein 4L	59.45	5.96%	2	94.5
17	16507237	78 kDa glucose-regulated protein precursor	0	3.52%	1	72.3
18	21536320	heterogeneous nuclear ribonucleoprotein U-like protein 1 isoform d	22.97	1.72%	1	84.7
19	4507677	endoplasmin precursor	209.51	20.55%	15	92.4
20	39780588	pre-rRNA-processing protein TSR1 homolog	0	1.12%	1	91.8
21	189458817	transferrin receptor protein 1	170.72	22.11%	14	84.8
22	79750824	niban-like protein 1 isoform 2	29.3	1.50%	1	82.6
23	4885375	histone H1.2	39.67	9.39%	2	21.4
24	33469919	DNA replication licensing factor MCM4	72.98	8.46%	6	96.5
25	25121987	condensin complex subunit 2	33.45	1.48%	1	82.5
26	295986608	immunoglobulin lambda-like polypeptide 5 isoform 1	193.47	40.65%	6	23
27	100816392	far upstream element-binding protein 3	48.09	1.40%	1	61.6
28	55770844	catenin alpha-1	0	1.21%	1	100

**Table 2:** Candidate 8 proteins selected as hepatocellular carcinoma associated antigens

No.	Accession no.	Identified proteins	Score	Unique peptides	Protein functions
1	12025678	alpha-actinin-4	210.42	29	Involved in tight junction assembly in epithelial cells
2	38569423	ATP-citrate synthase isoform 2	94.99	10	Enzyme for the synthesis of cytosolic acetyl-CoA
3	29029559	exportin-2 isoform 1	63.97	10	Export receptor for importin-alpha
4	4507677	endoplasmin precursor	209.5	15	Process and transport of secreted proteins
5	42544159	heat shock protein 105 kDa	100.27	9	Prevent the aggregation of denatured proteins in cells
6	38327039	heat shock 70 kDa protein 4	103.26	10	ATP binding
7	16507237	78 kDa glucose regulated protein precursor	1437.33	39	Facilitate the assembly of protein complexes inside the endoplasmic reticulum
8	440309857	heat shock protein 75 kDa	231.16	12	Chaperone that express an ATPase activity

### Detection of autoantibodies in sera from 173 HCC patients

**Figure 2:**
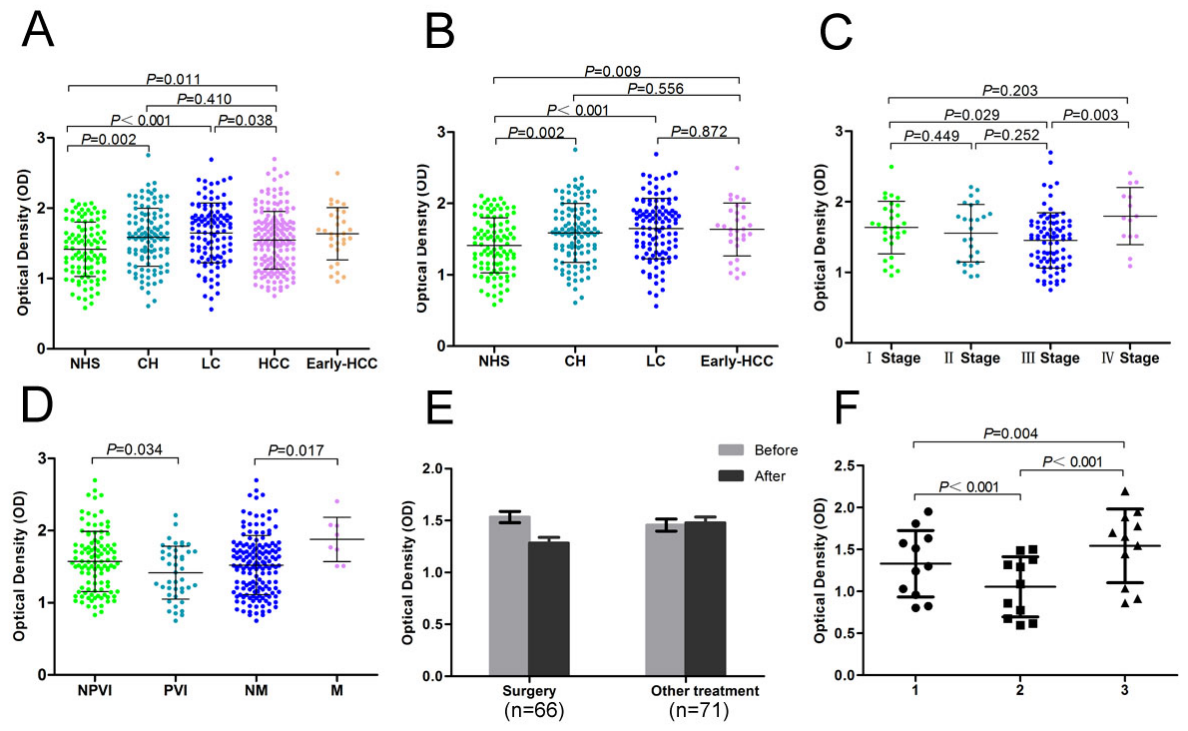
Titer of anti-GRP78 autoantibodies in human serum samples **A**. Comparison of the titer of anti-GRP78 autoantibodies in HCC with the other groups. **B**. Comparison the titer of anti-GRP78 autoantibodies in early-HCC with the other groups. **C**. Titer of anti-GRP78 autoantibodies in sera from HCC patients with different clinical stages. **D**. Titer of anti-GRP78 autoantibodies from HCC patients with and without portal vein invasion and metastasis. **E**. The comparision of anti-GRP78 autoantibodies in sera from HCC patients before treatment and within 1 week after treatment. **F**. The comparision of anti-GRP78 autoantibodies in sera from HCC patients with recurrence before surgery, within 1 week after surgery, and 1 month after surgery. GRP78, 78 kDa glucose regulated protein; NHS, normal human serum; CH, chronic hepatitis; LC, liver cirrhosis; HCC, hepatocellular carcinoma; NPVI, non-portal vein invasion; PVI, portal vein invasion; NM, non-metastasis; M, metastasis; 1, before surgery; 2, within 1 week after surgery; 3, 1 month after surgery.

Eight recombinant candidate proteins were commercially purchased and further used as coating antigens in ELISA for the detection of autoantibodies in sera from 173 HCC patients, 110 LC patients, 110 CH patients and 101control patients. Most autoantibodies against proteins such as GRP94, CSE1L, ACLY, GRP78, ACTN4, and HSPH1 had different expression levels when compared among HCC, LC, CH, and NHS groups (*P*<0.05). The expression of anti-GRP78 in HCC was higher than in the NHS (*P*<0.05) and in the early HCC compared to NHS group (Figure 2A). Compared with early-HCC, LC, CH, and NHS group, the titer of autoantibodies against GRP78 in early stage of HCC, LC, and CH groups were significantly higher than controls (Figure 2B). Table 3 demonstrates the frequency of serum autoantibodies against GRP78. The prevalence of anti-GRP78 autoantibodies in the HCC (*P*<0.01) and LC (*P*<0.01) groups was significantly higher than that in NHS groups. The sensitivity and specificity of anti-GRP78 autoantibody detection in HCC were 7.5% and 94.4%. Meanwhile, the positive predictive value and negative predict value of anti-GRP78 autoantibody detection in HCC were 41.9% and 65.4% (mean +2SD of NHS samples was used as a cutoff value). The titer of anti-GRP78 autoantibodies was different in sera of HCC patients at different stages of disease (*P*<0.01). The titer of anti-GRP78 autoantibodies showed a decreasing tendency in stage I, II, and III. The titer in sera from HCC patients with stage IV was higher than those in other stages (Figure 2C). The titer of anti-GRP78 autoantibodies correlated with clinical and pathological characteristics (both *P*<0.05) (Figure 2D). Autoantibody against GRP78 had no relationship with AFP. Interestingly, both AFP and anti-GRP78 autoantibody was only detected in sera of 1of 173 HCC patients. The concentration of AFP in 95 (55.2%) HCC patients was >200 ng/ml and >20 ng/ml in 129 (75.0%) HCC patients. When both AFP and anti-GRP78 autoantibody were used simultaneously as biomarkers, 98 (57%) HCC patients (AFP>200 ng/ml) and 132 (76.7%) HCC patients (AFP>20ng/ml) were positive, respectively.

**Table 3:** Frequency of autoantibodies against GRP78 in human serum samples by ELISA

Type of serum	No. tested	Frequency(patients with autoantibody/total patients with disease)
HCC	173	7.5% (13/173)**
LC	110	10.0% (11/110)**
CH	110	6.4% (7/110)
NHS	101	0% (0/101)

Cutoff value, mean + 2SD of NHS samples; **P <0.01, *P* value relative to NHS

Abbreviations: GRP78, 78 kDa glucose regulated protein; HCC, hepatocellular carcinoma; LC, liver cirrhosis; CH, chronic hepatitis; NHS, normal human serum.

### Validation of Anti-GRP78 antibodies as biomarkers for monitoring curative effects in HCC patients

The expression of anti-GRP78 autoantibodies in 137 HCC patients within 1 week after treatment was significantly decreased compared to HCC patients before treatment (P<0.001). There was no change in the expression of anti-GRP78 autoantibodies in patients without surgery (Figure 2E). The titer of anti-GRP78 autoantibodies was significantly higher 1 month after surgery in recurrent patients who accepted hepatic resection compared to patients before surgery or within 1 week after surgery (Figure 2F). There were no significant differences in the titer of anti-GRP78 autoantibodies in non-recurrent HCC patients. Eighteen (18) of 173 HCC patients were tested for AFP before and after 1 week of treatment. The concentration of AFP was significantly decreased within 1 week after treatment (P <0.001). Both the surgery and treatment groups showed decreased AFP, which was different from that of anti-GRP78 autoantibodies. AFP concentration in 13 recurrent HCC patients declined one month after surgery compared to before surgery (P<0.01). We found AFP declined in 9 recurrent HCC patients who had detectable levels of both AFP and GRP78 autoantibodies before surgery and 1 month after surgery while only the titer of autoantibodies against GRP78 was elevated 1 month after surgery.

### Intense perinuclear staining pattern detected in HeLa cells by indirect immunofluorescence assay with representative positive HCC serum

**Figure 3:**
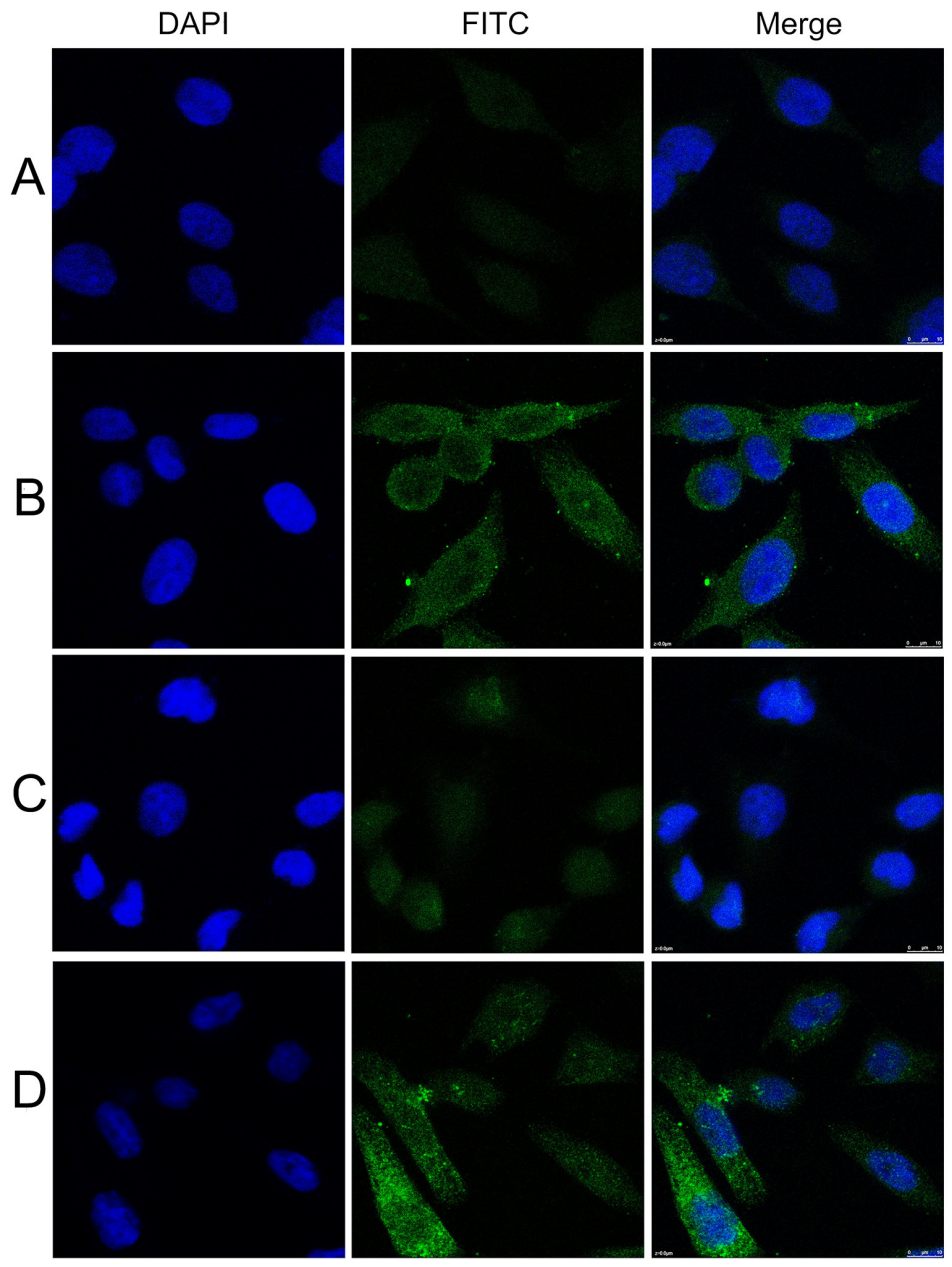
Representative immunofluorescence staining pattern of anti-GRP78 antibody positive HCC serum samples **A**. A normal human serum sample. **B**. A representative anti-GRP78 antibody positive HCC serum sample. **C**. The same HCC serum sample in B was pre-absorbed with recombinant GRP78 protein. **D**. Polyclonal anti-GRP78 antibody which showed a perinuclear immunofluorescence staining pattern was used as positive control. Abbreviations: GRP78, 78 kDa glucose regulated protein; HCC, hepatocellular carcinoma. (×63)

HCC serum with anti-GRP78 positive expression had a perinuclear staining pattern which was similar to that shown by polyclonal anti-GRP78 antibody (Figure 3). When the same HCC serum was pre-absorbed by recombinant GRP78 protein, the fluorescent staining was significantly reduced.

### Expression of GRP78 in different cancer cell lines

**Figure 4:**
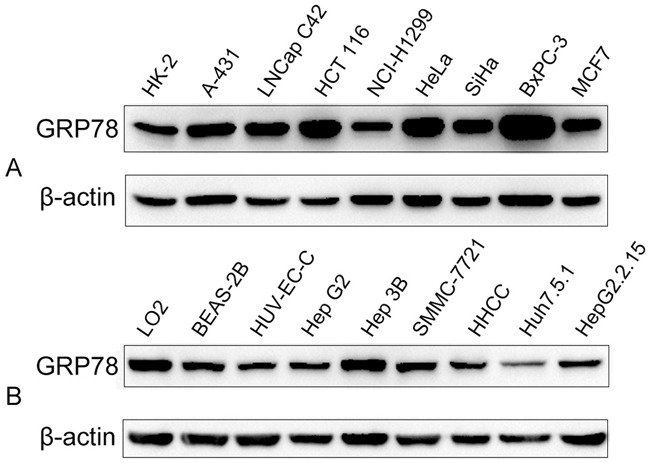
The expression of GRP78 in different cancer cell lines and the reaction of GRP78 with different serum samples **A**. The expression of GRP78 in other cancer cell lines. **B**. The expression of GRP78 in liver cancer cell lines.

HCT 116, BxPC-3, and LNCap C42 cell lines showed stronger reactive bands compared to HK-2, A-431, and NCI-H1299 cell lines when analyzed by Western blot (Figure 4A). The liver cancer cell lines Hep3B, HepG_2_, SMMC-7721, and liver cell line LO2 showed stronger GRP78 expression than HCC, Huh7.5.1, and HepG2.2.15 (Figure 4B).

### Expression of GRP78 in HCC tissues

**Figure 5:**
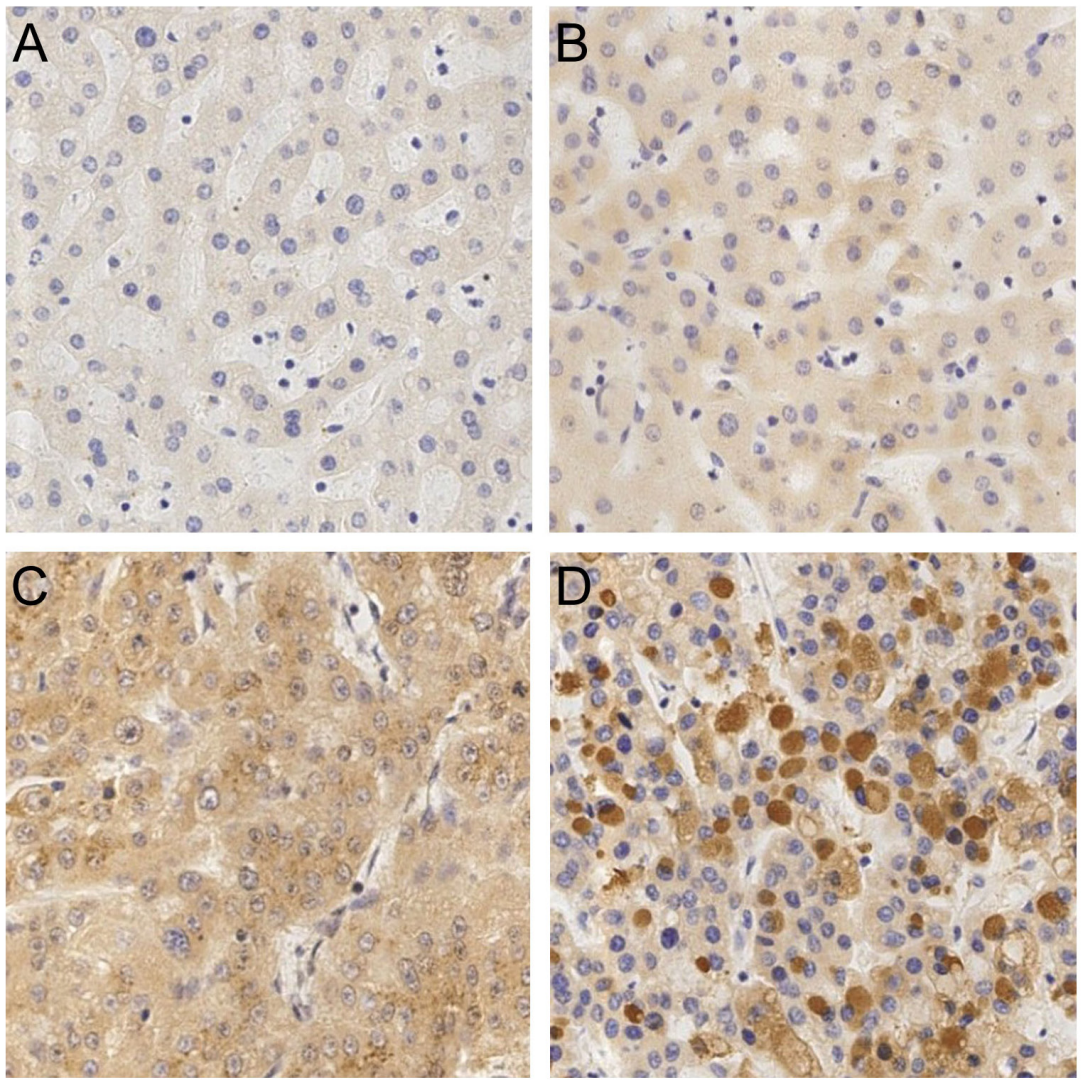
The expression of GRP78 in different liver tissues by immunohistochemistry **A**. a normal liver tissue. **B**. a liver tissue with chronic hepatitis. **C**. a liver tissue with liver cirrhosis. **D**. a liver tissue with hepatocellular carcinoma. (×20)

The expression of GRP78 in 38 HCC tissues, 18 LC tissues, 11 CH tissues and 4 normal liver tissues are shown in Figure 5. All HCC tissues (100.0%) were positive for GRP78 staining. GRP78-positive staining was observed in the cytoplasm of the cancer cells. Sixteen (16) of 18 LC, 9 of 11 CH and 3 of 4 normal liver tissues were positive for GRP78 staining. The positive rate of GRP78 in the HCC group was higher than in the non-HCC group (*P*<0.05). The expression of GRP78 in the HCC, LC, CH and normal liver tissues are shown in Table 4. In HCC, GRP78 was expressed in the high grades (28/38), while GRP78 in non-HCC group was mainly expressed in the low grades (23/33) (*P*<0.001). The expression of GRP78 had no correlation with clinical or pathological characteristics such as gender, tumor size, histological grade, clinical stage, capsular infiltration, portal vein invasion, lymphatic metastasis and distant metastasis.

**Table 4:** Expression of GRP78 in patients’ liver tissues with different diseases

Group	Expression of GRP78				*P*
	I(%)	II(%)	III(%)	IV(%)	
HCC	1 (2.6)	9 (23.7)	11 (28.9)	17 (44.7)	0.001
LC	2 (11.1)	10 (55.6)	5 (27.8)	1 (5.6)	0.005
CH	2 (18.2)	6 (54.5)	2 (18.2)	1 (9.1)	0.036
N	1 (25.0)	2 (50.0)	1 (25.0)	0 (0.0)	

Abbreviations: GRP78, 78 kDa glucose regulated protein; HCC, hepatocellular carcinoma; LC, liver cirrhosis; CH, chronic hepatitis; N, normal liver tissue; *P* value relative to normal liver tissue.

## DISCUSSION

GRP78, also known as immunoglobulin heavy chain binding protein (Bip) [17], belongs to the heat shock protein 70 family. GRP78 localizes to the endoplasmic reticulum (ER) and is involved in proper protein folding and assembly, proteosome degradation of misfolded proteins, ER Ca^2+^ binding, and the activation of transmembrane ER stress sensors [17, 18]. It is reported that deregulation of GRP78 has a causal relationship with tumor occurrence and progression [19–21]. The present study showed that the expression of GRP78 was higher in HCC than non-HCC groups, suggesting that GRP78 may be associated with HCC occurrence and progression. Zheng et al. [22] found that GRP78 expression in gastric carcinoma was positively linked to tumor size, depth of invasion, lymphatic and venous invasion, lymph node metastasis, and UICC staging. We did not find any relationship between the expression of GRP78 and clinicopathological features of HCC. This may be due to the limited number of specimens. This study revealed a high expression level of GRP78 in the LNCap C42, HCT 116, BxPC-3, and Hep 3B cancer cell lines and weak expression in the HK-2, NCI-H1299, Huh 7.5.1, and HUVEC cell lines. Whether GRP78 can be regarded as a biomarker for different types of cancer warrants further investigation.

Our results indicated that frequency of autoantibodies against GRP78 in sera from patients with HCC and LC were significantly higher than that in sera from normal controls. Further analysis indicated that the expression level of autoantibodies against GRP78 was associated with clinical stage, portal vein invasion, and metastasis. Interestingly, the titer of anti-GRP78 autoantibodies decreased from clinical stageIto stage III, but increased in the stage IV. The expression of anti-GRP78 autoantibodies increased in metastasis patients. One of the hallmarkers of cancer is the evasion of immune destruction [23], which may be associated with the above phenomenon. Intracellular proteins involved in carcinogenesis have been shown to provoke autoantibody responses [24–28]. We believe this to be one reason for the higher expression of anti-GRP78 in stage IV HCC.

Defresne et al. [29] found that an increase in GRP78 auto-antibody titer preceded the detection of a palpable tumor mass. This study showed the average titer of anti-GRP78 autoantibodies was higher in the early stage of HCC. Although our study showed the titer of autoantibodies against GRP78 in early-HCC, LC, and CH groups was significantly higher than NHS group, there was no difference among early-HCC, LC, and CH groups. The study also showed that the titer of anti-GRP78 autoantibodies in LC group was significantly higher than controls. We suggest TAA autoantibodies appear years before the detection of cancer. This is supported by a previous report [30]. The sensitivity and specitivity of anti-GRP78 autoantibody for HCC diagnosis were 7.5% and 94.4%, respectively. Both the prevalence of anti-GRP78 autoantibodies in HCC and LC groups were significantly higher than that in NHS groups. Furthermore, none of the 13 AFP negative HCC patients were anti-GRP78 autoantibody positive. Our results indicate that anti-GRP78 autoantibody may not be used for diagnosis of HCC because of its low sesitivity in HCC.

To compare changes in anti-GRP78 autoantibodies before and after treatments, HCC patients were divided into surgery and non-surgery groups. Anti-GRP78 autoantibodies decreased significantly in surgery group but no differences were observed in the non-surgery group. Previous reports found that chemotherapy and radiotherapy differentially affect the anti-GRP78 immune response. Moreover, radiation increased the concentration of GRP78 auto-Ab and chemotherapy reduced the concentration of GRP78 auto-Ab. Different treatments may have different effects on anti-TAA autoantibodies. We speculate that hepatic resection reduces tumor burden and reduces the production of GRP78 antigens and anti-GRP78 antibodies.

This study also showed that the expression level of anti-GRP78 autoantibodies differed before surgery and after surgery in recurrent HCC patients. The titer of anti-GRP78 autoantibodies before surgery was higher than that within 1 week after surgery, and lower than that1 month after surgery. The expression level of anti-GRP78 autoantibodies was detected in HCC serum at 1-15 months after surgery. Therefore, we speculated that the decrease then increase titer of anti-GRP78 autoantibodies after surgery may predict recurrence or metastasis. The fact that the titer of anti-GRP78 autoantibodies was more sensitive than AFP suggests that anti-GRP78 autoantibodies may serve as a potential marker for tumor recurrence and metastatic progression.

In conclusion, we identified a high level of anti-GRP78 autoantibodies in HCC and recurrent HCC patients, as well as a high expression of GRP78 in HCC. Due to similar titers of anti-GRP78 between liver cirrhosis group and HCC group, anti-GRP78 cannot be used as a diagnostic maker for HCC. The current data does not provide enough evidence to support GRP78 in carcinogenesis. We suggest that Anti-GRP78 autoantibody may be used to monitor metastasis of HCC, indicate recurrence after hepatic resection, and monitor the efficacy of surgery. Further studies with larger sample size and detailed pathological information are warranted.

## MATERIALS AND METHODS

### Patient characteristics

The number of total participants was 561, including 229 HCC, 119 LC, 110 CH, and 103 normal controls. 100 HCC patients, 30 LC patients, 30 CH patients, and 30 control patients were recruited to screen serum of HCC patients with high titers of autoantibodies against TAAs by Western blot (cohort 1). 173 HCC patients, 110 LC patients, 110 CH patients, and 101 control patients were recruited to detect the serum levels of autoantibodies against eight candidate TAAs (cohort 2). 137 HCC patients were recruited to detect the changes of autoantibodies against TAAs before and after treatment such as hepatic resection, liver transplantation, transcatheter arterial chemoembolization, radiofrequency ablation etc (cohort 3). This study was approved by the Ethics committee of The First Affiliated Hospital of Xi’an Jiaotong University.

### Serum samples and tissue specimens

Serum and liver tissue specimens from patients with HCC, LC, CH were collected from October 2011 to October 2013 in The First Affiliated Hospital of Xi’an Jiaotong University, Xi’an, Shannxi, China. HCC serum samples (cohort 1 and cohort 2) were collected before patients were treated with chemotherapy, radiotherapy or surgery. HCC serum samples from cohort 3 were collected before and after treatment. All blood samples were obtained by centrifugation at 3,000 rpm for 5 min and stored at -80°C.

### Cell culture and cell extracts

Eighteen cell lines, HepG2, Hep3B, SMMC-7721, HHCC, Huh7.5.1, HepG2.2.15, MCF7, BxPC-3, HeLa, SiHa, HCT 116, NCI-H1299, LNCap C42, A-431, HK-2, LO2, BEAS-2B, and HUVEC were obtained from the Department of Transformation Medical Center of Xi’an Jiaotong University.

### Western blotting

HepG2, NCI-H1299, HeLa, and MCF7 cancer cell lysates were electrophoresed on 10% sodium dodecyl sulfate polyacrylamide gel electropheresis (SDS-PAGE) and transferred to polyvinylidene fluoride (PVDF) membranes to screen the autoantibody-positive serum. The membranes were incubated with 1:100 dilution of human serum for 8 h, and then incubated with horseradish peroxidase (HRP)-conjugated goat anti-human IgG (1:2000 dilution) (Abcam Inc, Cambridge, MA, USA). An enhanced chemiluminescence kit (Millipore Corporation, Billerica, MA, USA) was used to detect immunoreactive bands.

### Immunoprecipitation

HepG2 Cells extracts were prepared by re-suspending cells in 1.5 ml of radioimmunoprecipitation assay (RIPA) lysis buffer, sonicating for 5 min and centrifuging at 14,000 rpm for 10min at 4 °C. The HepG2 antigen(s) were immunoprecipitated from these samples using the IP Kit (Invitrogen, USA) according to manufacturer's instructions. Briefly, antibodies from HCC serum was immobilised on dynabeads. The antigen (whole cell extracts of HepG2 cells) was added to immobilised antibody gel mixture together with binding buffer on a rocking platform at room temperature. The antigens were eluted from the beads into the elution buffer using microcentrifuge spin cups.

### SDS-PAGE electrophoresis and in-Gel digestion

Following gel electrophoresis, gels were stained with Brilliant Blue Colloidal Coomassie to visualise precipitated proteins. Target gels were excised from the gel and destained with 0.4 mL of 100 mM NH_4_HCO_3_/30% acetonitrile (ACN). Proteins were in-gel reduced with 100 mM DTT for 30 min at 56°C and S-alkylated with 200 mM iodoacetamide for 20 min in the dark. Gel particles were washed with 100 mM NH_4_HCO_3_ and dehydrated for 5 min at room temperature in 100% ACN, then dried by Speed Vac. Trypsin Gold solution (Promega, WI) was used to rehydrate gel at 37°C for 20 h. All extracts were collected in a new microcentrifuge tube and dried by Speed Vac. The tryptic peptides were resuspended in 0.1% formic acid for LC-MS/MS analysis.

### Liquid chromatography-tandem mass spectrometry (LC-MS/MS) analysis

After in-Gel Digestion, the freeze-dried samples were resuspended in 0.1% formic acid and desalted by C18 peptide traps. Peptides were separated in a C18 capillary column (3.5 mm, 300 Å, Agilent Technologies, Santa Clara, CA). Eluted peptides were subjected to LC-MS/MS analysis, using nanoelectrospray ionization (TriVersa NanoMate system, Advion) coupled to a linear ion trap-mass specmeter (LTQ XL, Thermo-Fisher Scientific). All MS/MS data were searched against the human protein database from National Center for Biotechnology Information (NCBI) nonredundant database by Bioworks version 3.3 software (SEQUEST, Thermo Electron) installed on a local server. SEQUEST filter criteria were used as follows: charge = 1, Xcorr ≥ 1.9; charge = 2, Xcorr ≥ 2.2; charge = 3, Xcorr ≥ 3.75; DeltaCn ≥ 0.25.

### Bioinformatics analysis

Bioinformation was obtained from websites such as http://www.uniprot.org, http://ncbi.nlm.nih.gov, http://geneontology.org, http://david.abcc.ncifcrf.gov, and http://www.genome.jp/kegg. Target proteins were selected based on the following criteria: 1. An ion score cutoff of 20 to ensure the quality of valid peptides and to remove redundant protein hits, and the uniquepeptides ≥2; 2. Select proteins close to the target molecular weight; 3. Protein functions associated with tumor.

### ELISA

96-well microtiter plates were coated overnight at 4°C with recombinant proteins (Proteintech Group Inc, Chicago, IL, USA) at a final concentration of 1.0 μg/ml. Human serum (1:80 diluted in 1% BSA/PBS) was incubated for 2 h at 37°C. HRP-conjugated goat anti-human IgG (1:4000 diluted) (Abcam Inc, Cambridge, MA, USA) and tetramethylbenzidine (TMB) were used as detecting reagents. The optical density (OD) value at a wavelength of 450 nm was applied.

### Immunohistochemistry

Specimens of 38 HCC tissues, 18 LC tissues, 11 CH tissues, and 4 normal liver tissue were deparaffinized and rehydrated. The sections were incubated with polyclonal anti-GRP78 antibody (1:100 dilution) (Proteintech Group, USA). HRP detection system (HRP streptavidin labeled and polyvalent biotinylated linked) and DAB substrate kit were used as detecting reagents (Zhongshanjinqiao, China). Two independent pathologists evaluated and scored the IHC staining. Scoring of cytoplasmic GRP78 staining was evaluated as previously reported [16].

### Absorption of antibodies with recombinant protein

Serum (1:25 dilution) was incubated with recombinant GRP78 protein overnight at 4°C, and then centrifuged at 10,000 x g for 15 min. The final concentration of the protein diluted with HCC serum was 0.01 μg/μl. The supernatant was used for immunofluorescence assay.

### Indirect immunofluorescence

Indirect immunofluorescence was performed to confirm the reactivity of anti-GRP78 autoantibodies in HCC serum samples and the intracellular location of GRP78. The diluted human sera (1:25) was incubated with recombinant protein (final concentration of recombinant protein in the diluted human sera was 0.01μg/μl) overnight at 4C, then centrifuged at 10,000xg for 10min. The supernatant was used for indirect immunofluorescence assay.

HeLa cells were incubated with serum (1:25 dilution) and preabsorbed serum (1:25 dilution) overnight at 4°C. FITC-conjugated goat anti-human IgG (Proteintech Group Inc, Chicago, IL, USA) was used as the secondary antibody at a 1:20 dilution. Fluorescence microscope (Leica DM1000, Germany) was used for examination.

### Statistics

SPSS version 18.0 (SPSS Inc., Chicago, IL) was used to analyze the data. The mean OD value of autoantibodies against candidate proteins in each group was compared using analysis of variance. The autoantibody frequency to GRP78 of patients’ sera in each group was compared by Chi-Square test and fisher's exact test. Comparison of the clinicopathological parameters with GRP78 expression was conducted by the two-tail Mann-Whitney U-test. The level of autoantibodies against GRP78 before and after treatment and AFP were compared by the means of the randomized block design ANOVA and rank sum test. A *P*<0.05 were considered to be statistically significant.

## SUPPLEMENTARY MATERIALS TABLES




