# Supplementary material for: Autoantibodies against glucose-regulated protein 78 as serological biomarkers in metastatic and recurrent hepatocellular carcinoma
Source: Oncotarget. 2017 Feb 8;8(15):24828–39. doi: 10.18632/oncotarget.15192 (PMC5421892; doi:10.18632/oncotarget.15192)
Supplement: Supplementary file 1 [file oncotarget-08-24828-s001.pdf]

Autoantibodies against glucose-regulated protein 78 as serological biomarkers in metastatic and recurrent hepatocellular carcinoma

SUPPLEMENTARY TABLES

Supplementary Table 1: Frequency of autoantibodies against the autoantigens in sera from patients with hepatocellular carcinoma, liver cirrhosis, chronic hepatitis, and sera from normal human individuals

molecular weight (kDa)	frequency			
	HCC	LC	CH	NHS
113	11% (11/100)	-	-	3% (1/30)
112	14% (14/100)	-	10% (3/30)	3% (1/30)
98	12% (12/100)	3% (1/30)	10% (3/30)	3% (1/30)
76	14% (14/100)	-	3% (1/30)	10% (3/30)

-, no detected; HCC, hepatocellular carcinoma; LC, liver cirrhosis; CH, chronic hepatitis; NHS, normal human serum.

Supplementary Table 2: The identified 566 proteins by proteomic approach.

See Supplementary File 1

Supplementary Table 3: Clinicopathological characteristics of recruited participants (n=494)

Variable	HCC	N	LC	CH	*p*
	n=173	n=101	n=110	n=110	
Age					<0.001
Mean	52.84	52.72	51.75	47.05	
Range	26-78	22-86	32-82	24-79	
Sex, n(%)					0.756
Male	145 (83.8%)	83 (82.2%)	92 (83.6%)	87 (79.1%)	
Female	28 (16.2%)	18 (17.8%)	18 (16.4%)	23 (20.9%)	
TNM stage(AJCC)					
I	30 (17.3%)				
II	25 (14.5%)				
III	85 (49.1%)				
IV	14 (8.1%)				
Missing	19 (11.0%)				
histological grade					
I	5 (2.9%)				
II	79 (45.7%)				
III	37 (21.4%)				
Missing	52 (30.1%)				
Tumor size					
≤3 cm	28 (16.2%)				
≤3 cm	95 (54.9%)				
Missing	50 (28.9%)				
Capsular infiltration					
Negtive	26 (15.0%)				
Positive	83 (48.0%)				
Missing	64 (37.0%)				
portal vein invasion					
Negtive	106 (61.3%)				
Positive	43 (24.9%)				
Missing	24 (13.9%)				
metastasis					
Negtive	156 (90.2%)				
Positive	8 (4.6%)				
Missing	9 (5.2%)				

HCC, hepatocellular carcinoma; LC, liver cirrhosis; CH, chronic hepatitis; N, normal controls; TNM, T, Tumor; N, Node; M, Metastasis; AJCC, American Joint Committee on Cancer Staging;
