# Supplementary material for: Autoantibodies against glucose-regulated protein 78 as serological biomarkers in metastatic and recurrent hepatocellular carcinoma
Source: Oncotarget. 2017 Feb 8;8(15):24828–39. doi: 10.18632/oncotarget.15192 (PMC5421892; doi:10.18632/oncotarget.15192)
Supplement: Supplementary file 2 [file oncotarget-08-24828-s002.docx]

**Supplementary Table 2: The identified 566 proteins by Proteomic Approach**

| **A Accession** | **Description** | **Score** | **Coverage** | **# Proteins** | **# Unique Peptides** | **# Peptides** | **# PSMs** | **Area** | **# AAs** | **MW [kDa]** | **calc. pI** |
| --- | --- | --- | --- | --- | --- | --- | --- | --- | --- | --- | --- |
| 4501867 | aconitate hydratase, mitochondrial precursor [Homo sapiens] | 153.8 | 14.36% | 1 | 8 | 8 | 8 |  | 780 | 85.4 | 7.61 |
| 4502011 | adenylate kinase isoenzyme 1 [Homo sapiens] | 0 | 1.88% | 5 | 1 | 1 | 1 |  | 799 | 88.2 | 8.27 |
| 4502027 | serum albumin preproprotein [Homo sapiens] | 199.6 | 27.09% | 1 | 17 | 17 | 17 |  | 609 | 69.3 | 6.28 |
| 4502067 | protein AMBP preproprotein [Homo sapiens] | 0 | 7.67% | 1 | 2 | 2 | 2 |  | 352 | 39 | 6.25 |
| 4502101 | annexin A1 [Homo sapiens] | 106.73 | 34.10% | 1 | 9 | 9 | 9 |  | 346 | 38.7 | 7.02 |
| 4502205 | ADP-ribosylation factor 4 [Homo sapiens] | 0 | 8.33% | 5 | 2 | 2 | 2 |  | 180 | 20.5 | 7.14 |
| 4502337 | zinc-alpha-2-glycoprotein precursor [Homo sapiens] | 0 | 3.36% | 1 | 1 | 1 | 1 |  | 298 | 34.2 | 6.05 |
| 4502549 | calmodulin [Homo sapiens] | 43.98 | 13.42% | 3 | 2 | 2 | 2 |  | 149 | 16.8 | 4.22 |
| 4503105 | cystatin-SA precursor [Homo sapiens] | 0 | 4.96% | 2 | 1 | 1 | 1 |  | 141 | 16.4 | 4.93 |
| 4503117 | cystatin-B [Homo sapiens] | 34.9 | 24.49% | 1 | 2 | 2 | 2 |  | 98 | 11.1 | 7.56 |
| 4503471 | elongation factor 1-alpha 1 [Homo sapiens] | 286.92 | 28.14% | 1 | 3 | 11 | 14 |  | 462 | 50.1 | 9.01 |
| 4503475 | elongation factor 1-alpha 2 [Homo sapiens] | 194.99 | 22.03% | 1 | 2 | 10 | 12 |  | 463 | 50.4 | 9.03 |
| 4503481 | elongation factor 1-gamma [Homo sapiens] | 39.67 | 2.97% | 1 | 1 | 1 | 1 |  | 437 | 50.1 | 6.67 |
| 4503483 | elongation factor 2 [Homo sapiens] | 202.81 | 33.80% | 4 | 26 | 26 | 33 |  | 858 | 95.3 | 6.83 |
| 4503507 | eukaryotic translation initiation factor 2 subunit 3 [Homo sapiens] | 28.65 | 4.03% | 1 | 2 | 2 | 2 |  | 472 | 51.1 | 8.4 |
| 4503525 | eukaryotic translation initiation factor 3 subunit C [Homo sapiens] | 0 | 1.10% | 3 | 1 | 1 | 1 |  | 913 | 105.3 | 5.68 |
| 4503529 | eukaryotic initiation factor 4A-I isoform 1 [Homo sapiens] | 102.48 | 12.07% | 3 | 4 | 4 | 4 |  | 406 | 46.1 | 5.48 |
| 4503571 | alpha-enolase isoform 1 [Homo sapiens] | 39.6 | 5.76% | 5 | 2 | 2 | 2 |  | 434 | 47.1 | 7.39 |
| 4504035 | GMP synthase [glutamine-hydrolyzing] [Homo sapiens] | 140.24 | 17.03% | 1 | 10 | 10 | 11 |  | 693 | 76.7 | 6.87 |
| 4504061 | N-acetylglucosamine-6-sulfatase precursor [Homo sapiens] | 0 | 6.16% | 1 | 3 | 3 | 3 |  | 552 | 62 | 8.31 |
| 4504183 | glutathione S-transferase P [Homo sapiens] | 0 | 9.52% | 1 | 1 | 1 | 1 |  | 210 | 23.3 | 5.64 |
| 4504303 | histone H4 [Homo sapiens] | 30.03 | 27.18% | 3 | 3 | 3 | 3 |  | 103 | 11.4 | 11.36 |
| 4504345 | hemoglobin subunit alpha [Homo sapiens] | 46.23 | 19.72% | 2 | 3 | 3 | 3 |  | 142 | 15.2 | 8.68 |
| 4504349 | hemoglobin subunit beta [Homo sapiens] | 59.47 | 28.57% | 2 | 3 | 3 | 3 |  | 147 | 16 | 7.28 |
| 4504505 | peroxisomal multifunctional enzyme type 2 isoform 2 [Homo sapiens] | 25.93 | 5.43% | 3 | 3 | 3 | 3 |  | 736 | 79.6 | 8.84 |
| 4504517 | heat shock protein beta-1 [Homo sapiens] | 95.93 | 30.24% | 1 | 5 | 5 | 5 |  | 205 | 22.8 | 6.4 |
| 4504957 | lysosome-associated membrane glycoprotein 2 isoform A precursor [Homo sapiens] | 61.24 | 2.93% | 3 | 1 | 1 | 1 |  | 410 | 44.9 | 5.63 |
| 4505257 | moesin [Homo sapiens] | 1058.77 | 61.18% | 1 | 35 | 45 | 63 |  | 577 | 67.8 | 6.4 |
| 4505343 | nuclear cap-binding protein subunit 1 [Homo sapiens] | 32.64 | 6.96% | 1 | 5 | 5 | 5 |  | 790 | 91.8 | 6.43 |
| 4505541 | general vesicular transport factor p115 [Homo sapiens] | 0 | 0.94% | 1 | 1 | 1 | 1 |  | 962 | 107.8 | 4.91 |
| 4505891 | procollagen-lysine,2-oxoglutarate 5-dioxygenase 3 precursor [Homo sapiens] | 105.36 | 18.29% | 1 | 11 | 11 | 11 |  | 738 | 84.7 | 6.05 |
| 4505917 | exosome component 10 isoform 2 [Homo sapiens] | 40.16 | 1.98% | 2 | 2 | 2 | 2 |  | 860 | 98 | 8.4 |
| 4506025 | calcineurin subunit B type 1 [Homo sapiens] | 488.08 | 28.45% | 17 | 16 | 23 | 36 |  | 717 | 79.4 | 4.94 |
| 4506113 | major prion protein preproprotein [Homo sapiens] | 34.17 | 3.32% | 6 | 4 | 4 | 4 |  | 1386 | 157.2 | 7.36 |
| 4506411 | ran GTPase-activating protein 1 [Homo sapiens] | 120.57 | 19.42% | 1 | 9 | 9 | 9 |  | 587 | 63.5 | 4.68 |
| 4506467 | radixin isoform 2 [Homo sapiens] | 398.61 | 35.16% | 5 | 14 | 26 | 31 |  | 583 | 68.5 | 6.37 |
| 4506623 | 60S ribosomal protein L27 [Homo sapiens] | 0 | 2.43% | 1 | 1 | 1 | 1 |  | 371 | 42.9 | 10.51 |
| 4506629 | 60S ribosomal protein L29 [Homo sapiens] | 58.12 | 14.47% | 1 | 2 | 2 | 2 |  | 159 | 17.7 | 11.66 |
| 4506661 | 60S ribosomal protein L7a [Homo sapiens] | 32.13 | 16.17% | 1 | 4 | 4 | 4 |  | 266 | 30 | 10.61 |
| 4506675 | dolichyl-diphosphooligosaccharide--protein glycosyltransferase subunit 1 precursor [Homo sapiens] | 38.02 | 1.98% | 1 | 1 | 1 | 1 |  | 607 | 68.5 | 6.38 |
| 4506691 | 40S ribosomal protein S16 [Homo sapiens] | 39.92 | 6.85% | 1 | 1 | 1 | 1 |  | 146 | 16.4 | 10.21 |
| 4506743 | 40S ribosomal protein S8 [Homo sapiens] | 32.07 | 9.62% | 1 | 2 | 2 | 2 |  | 208 | 24.2 | 10.32 |
| 4506749 | ribonucleoside-diphosphate reductase large subunit [Homo sapiens] | 168.3 | 17.42% | 1 | 12 | 12 | 13 |  | 792 | 90 | 7.15 |
| 4506773 | protein S100-A9 [Homo sapiens] | 27.91 | 9.62% | 3 | 4 | 4 | 4 |  | 468 | 54.2 | 9.67 |
| 4507115 | fascin [Homo sapiens] | 0 | 2.43% | 1 | 1 | 1 | 1 |  | 493 | 54.5 | 7.24 |
| 4507241 | FACT complex subunit SSRP1 [Homo sapiens] | 191.65 | 21.02% | 1 | 13 | 13 | 13 |  | 709 | 81 | 6.87 |
| 4507491 | thimet oligopeptidase [Homo sapiens] | 104.92 | 12.34% | 1 | 8 | 8 | 8 |  | 689 | 78.8 | 6.05 |
| 4507521 | transketolase isoform 1 [Homo sapiens] | 33.34 | 7.54% | 2 | 4 | 4 | 4 |  | 623 | 67.8 | 7.66 |
| 4507555 | thymopoietin isoform alpha [Homo sapiens] | 140.97 | 10.81% | 3 | 5 | 5 | 5 |  | 694 | 75.4 | 7.66 |
| 4507677 | endoplasmin precursor [Homo sapiens] | 176.54 | 27.27% | 1 | 18 | 20 | 20 |  | 803 | 92.4 | 4.84 |
| 4507877 | vinculin isoform VCL [Homo sapiens] | 79.75 | 11.44% | 2 | 9 | 9 | 9 |  | 1066 | 116.6 | 6.09 |
| 4507943 | exportin-1 [Homo sapiens] | 111.92 | 17.93% | 1 | 15 | 15 | 16 |  | 1071 | 123.3 | 6.06 |
| 4507953 | 14-3-3 protein zeta/delta [Homo sapiens] | 57.26 | 29.80% | 2 | 4 | 6 | 6 |  | 245 | 27.7 | 4.79 |
| 4557014 | catalase [Homo sapiens] | 62.31 | 2.47% | 1 | 1 | 1 | 1 |  | 527 | 59.7 | 7.39 |
| 4557305 | fructose-bisphosphate aldolase A isoform 1 [Homo sapiens] | 0 | 2.20% | 2 | 1 | 1 | 1 |  | 364 | 39.4 | 8.09 |
| 4557321 | apolipoprotein A-I preproprotein [Homo sapiens] | 28 | 3.00% | 1 | 1 | 1 | 1 |  | 267 | 30.8 | 5.76 |
| 4557369 | bone morphogenetic protein 2 preproprotein [Homo sapiens] | 84.29 | 15.86% | 7 | 3 | 7 | 12 |  | 851 | 95.9 | 7.15 |
| 4557469 | AP-2 complex subunit beta isoform b [Homo sapiens] | 56.89 | 6.40% | 5 | 5 | 5 | 5 |  | 937 | 104.5 | 5.38 |
| 4557471 | AP-1 complex subunit sigma-1A [Homo sapiens] | 249.7 | 26.54% | 8 | 5 | 9 | 31 |  | 599 | 68.1 | 5.55 |
| 4557581 | fatty acid-binding protein, epidermal [Homo sapiens] | 34.35 | 6.67% | 1 | 1 | 1 | 1 |  | 135 | 15.2 | 7.01 |
| 4557701 | keratin, type I cytoskeletal 17 [Homo sapiens] | 201.55 | 18.29% | 13 | 2 | 10 | 13 |  | 432 | 48.1 | 5.02 |
| 4557733 | latent-transforming growth factor beta-binding protein 2 precursor [Homo sapiens] | 27.82 | 0.44% | 1 | 1 | 1 | 1 |  | 1821 | 194.9 | 5.19 |
| 4557871 | serotransferrin precursor [Homo sapiens] | 0 | 1.43% | 1 | 1 | 1 | 1 |  | 698 | 77 | 7.12 |
| 4557894 | lysozyme C precursor [Homo sapiens] | 58.87 | 10.81% | 1 | 2 | 2 | 2 |  | 148 | 16.5 | 9.16 |
| 4757900 | calreticulin precursor [Homo sapiens] | 0 | 2.16% | 1 | 1 | 1 | 1 |  | 417 | 48.1 | 4.44 |
| 4758032 | coatomer subunit beta' [Homo sapiens] | 0 | 0.99% | 1 | 1 | 1 | 1 |  | 906 | 102.4 | 5.27 |
| 4758304 | protein disulfide-isomerase A4 precursor [Homo sapiens] | 473.83 | 35.81% | 1 | 21 | 21 | 26 |  | 645 | 72.9 | 5.07 |
| 4758332 | long-chain-fatty-acid--CoA ligase 4 isoform 1 [Homo sapiens] | 28.67 | 4.93% | 3 | 2 | 2 | 2 |  | 670 | 74.4 | 8.03 |
| 4758528 | hepatocyte growth factor-regulated tyrosine kinase substrate [Homo sapiens] | 22.99 | 1.54% | 1 | 1 | 1 | 1 |  | 777 | 86.1 | 6.16 |
| 4758556 | U4/U6 small nuclear ribonucleoprotein Prp3 [Homo sapiens] | 53.03 | 2.34% | 1 | 2 | 2 | 2 |  | 683 | 77.5 | 9.5 |
| 4758638 | peroxiredoxin-6 [Homo sapiens] | 0 | 4.02% | 1 | 1 | 1 | 1 |  | 224 | 25 | 6.38 |
| 4758648 | kinesin-1 heavy chain [Homo sapiens] | 35.74 | 12.56% | 3 | 10 | 10 | 10 |  | 963 | 109.6 | 6.51 |
| 4758920 | phosphatidylinositol N-acetylglucosaminyltransferase subunit H [Homo sapiens] | 39.2 | 3.70% | 2 | 1 | 2 | 2 |  | 487 | 54.9 | 5.64 |
| 4759050 | ribosomal protein S6 kinase alpha-3 [Homo sapiens] | 140.88 | 10.14% | 17 | 3 | 8 | 9 |  | 740 | 83.7 | 6.89 |
| 4826686 | ATP-dependent RNA helicase DDX1 [Homo sapiens] | 128.32 | 23.51% | 1 | 15 | 15 | 15 |  | 740 | 82.4 | 7.23 |
| 4826702 | desmocollin-1 isoform Dsc1b preproprotein [Homo sapiens] | 40.67 | 1.79% | 2 | 1 | 1 | 1 |  | 840 | 93.8 | 5.53 |
| 4826998 | splicing factor, proline- and glutamine-rich [Homo sapiens] | 53.25 | 3.96% | 3 | 3 | 3 | 3 |  | 707 | 76.1 | 9.44 |
| 4885111 | calmodulin-like protein 3 [Homo sapiens] | 41.04 | 16.78% | 1 | 2 | 2 | 2 |  | 149 | 16.9 | 4.42 |
| 4885165 | cystatin-A [Homo sapiens] | 43.63 | 19.39% | 1 | 2 | 2 | 2 |  | 98 | 11 | 5.5 |
| 4885375 | histone H1.2 [Homo sapiens] | 58.64 | 18.78% | 5 | 3 | 3 | 3 |  | 213 | 21.4 | 10.93 |
| 4885607 | small proline-rich protein 3 [Homo sapiens] | 26.47 | 14.20% | 1 | 2 | 2 | 2 |  | 169 | 18.1 | 8.57 |
| 5031755 | heterogeneous nuclear ribonucleoprotein R isoform 2 [Homo sapiens] | 151.22 | 16.43% | 4 | 7 | 9 | 9 |  | 633 | 70.9 | 8.13 |
| 5031857 | L-lactate dehydrogenase A chain isoform 1 [Homo sapiens] | 39.2 | 10.54% | 5 | 3 | 3 | 3 |  | 332 | 36.7 | 8.27 |
| 5031863 | galectin-3-binding protein precursor [Homo sapiens] | 0 | 1.71% | 1 | 1 | 1 | 1 |  | 585 | 65.3 | 5.27 |
| 5031945 | helix-loop-helix protein 2 [Homo sapiens] | 239.14 | 5.81% | 7 | 5 | 13 | 17 |  | 2185 | 247.6 | 5.62 |
| 5032057 | protein S100-A11 [Homo sapiens] | 0 | 15.24% | 1 | 1 | 1 | 1 |  | 105 | 11.7 | 7.12 |
| 5032087 | splicing factor 3A subunit 1 isoform 1 [Homo sapiens] | 55.9 | 7.31% | 2 | 6 | 6 | 7 |  | 793 | 88.8 | 5.22 |
| 5032179 | transcription intermediary factor 1-beta [Homo sapiens] | 107.24 | 15.69% | 1 | 9 | 9 | 10 |  | 835 | 88.5 | 5.77 |
| 5174457 | kinetochore protein NDC80 homolog [Homo sapiens] | 23.6 | 2.65% | 1 | 2 | 2 | 2 |  | 642 | 73.9 | 5.6 |
| 5453710 | LIM and SH3 domain protein 1 [Homo sapiens] | 0 | 1.00% | 5 | 1 | 1 | 1 |  | 1199 | 138.1 | 7.18 |
| 5453860 | cyclin-dependent kinase 16 isoform 1 [Homo sapiens] | 0 | 1.61% | 3 | 1 | 1 | 1 |  | 496 | 55.7 | 7.62 |
| 5453998 | importin-7 [Homo sapiens] | 64.28 | 7.80% | 1 | 6 | 6 | 6 |  | 1038 | 119.4 | 4.82 |
| 5454052 | 14-3-3 protein sigma [Homo sapiens] | 53.24 | 21.37% | 6 | 4 | 6 | 6 |  | 248 | 27.8 | 4.74 |
| 5454168 | ranBP-type and C3HC4-type zinc finger-containing protein 1 isoform 1 [Homo sapiens] | 0 | 2.78% | 2 | 1 | 1 | 1 |  | 468 | 52.9 | 5.54 |
| 5729877 | heat shock cognate 71 kDa protein isoform 1 [Homo sapiens] | 272.21 | 28.02% | 3 | 11 | 15 | 17 |  | 646 | 70.9 | 5.52 |
| 5803181 | stress-induced-phosphoprotein 1 [Homo sapiens] | 45.89 | 2.39% | 1 | 1 | 1 | 1 |  | 543 | 62.6 | 6.8 |
| 6005830 | plakophilin-3 [Homo sapiens] | 100.52 | 7.53% | 1 | 5 | 5 | 5 |  | 797 | 87 | 9.32 |
| 6005942 | transitional endoplasmic reticulum ATPase [Homo sapiens] | 142.55 | 20.72% | 2 | 14 | 14 | 14 |  | 806 | 89.3 | 5.26 |
| 6274552 | signal transducer and activator of transcription 1-alpha/beta isoform alpha [Homo sapiens] | 56.66 | 15.07% | 2 | 10 | 10 | 12 |  | 750 | 87.3 | 6.05 |
| 6631095 | DNA replication licensing factor MCM3 [Homo sapiens] | 63.65 | 6.81% | 1 | 5 | 5 | 5 |  | 808 | 90.9 | 5.77 |
| 6912286 | caspase-14 precursor [Homo sapiens] | 0 | 5.37% | 1 | 1 | 1 | 1 |  | 242 | 27.7 | 5.58 |
| 6912482 | LETM1 and EF-hand domain-containing protein 1, mitochondrial precursor [Homo sapiens] | 86.23 | 11.23% | 1 | 7 | 7 | 7 |  | 739 | 83.3 | 6.7 |
| 6912486 | U6 snRNA-associated Sm-like protein LSm4 isoform 1 [Homo sapiens] | 22.81 | 5.04% | 1 | 1 | 1 | 1 |  | 139 | 15.3 | 9.99 |
| 7427519 | DNA replication licensing factor MCM6 [Homo sapiens] | 85.89 | 9.38% | 1 | 6 | 6 | 6 |  | 821 | 92.8 | 5.41 |
| 7657069 | ERO1-like protein alpha precursor [Homo sapiens] | 0 | 2.56% | 1 | 1 | 1 | 1 |  | 468 | 54.4 | 5.68 |
| 7657138 | Golgi integral membrane protein 4 [Homo sapiens] | 28.01 | 5.32% | 1 | 3 | 3 | 3 |  | 696 | 81.8 | 4.77 |
| 7657581 | calcium-binding mitochondrial carrier protein Aralar2 isoform 2 [Homo sapiens] | 0 | 2.52% | 3 | 2 | 2 | 2 |  | 675 | 74.1 | 8.62 |
| 7669492 | glyceraldehyde-3-phosphate dehydrogenase isoform 1 [Homo sapiens] | 99.21 | 22.39% | 2 | 5 | 5 | 5 |  | 335 | 36 | 8.46 |
| 7705369 | coatomer subunit beta [Homo sapiens] | 30.74 | 2.20% | 1 | 2 | 2 | 2 |  | 953 | 107.1 | 6.05 |
| 7706427 | cleavage and polyadenylation specificity factor subunit 3 [Homo sapiens] | 0 | 1.32% | 1 | 1 | 1 | 1 |  | 684 | 77.4 | 5.6 |
| 7706501 | WW domain-binding protein 11 [Homo sapiens] | 31.31 | 1.72% | 1 | 1 | 1 | 1 |  | 641 | 70 | 8.38 |
| 7706635 | cornulin [Homo sapiens] | 41.54 | 10.71% | 1 | 4 | 4 | 4 |  | 495 | 53.5 | 6.1 |
| 7706706 | sorting nexin-9 [Homo sapiens] | 0 | 1.18% | 1 | 1 | 1 | 1 |  | 595 | 66.5 | 5.58 |
| 8393009 | vacuolar protein sorting-associated protein 51 homolog [Homo sapiens] | 0 | 1.02% | 1 | 1 | 1 | 1 |  | 782 | 86 | 6.47 |
| 8659555 | cytoplasmic aconitate hydratase [Homo sapiens] | 0 | 1.01% | 1 | 1 | 1 | 1 |  | 889 | 98.3 | 6.68 |
| 8922301 | WD repeat-containing protein 70 [Homo sapiens] | 28.87 | 1.68% | 1 | 1 | 1 | 1 |  | 654 | 73.2 | 6.33 |
| 8923904 | bridging integrator 3 [Homo sapiens] | 0 | 5.14% | 1 | 1 | 1 | 1 |  | 253 | 29.6 | 7.47 |
| 9966799 | something about silencing protein 10 [Homo sapiens] | 0 | 1.88% | 1 | 1 | 1 | 1 |  | 479 | 54.5 | 5.62 |
| 9966805 | ATP-dependent RNA helicase DDX24 [Homo sapiens] | 24.04 | 1.28% | 1 | 1 | 1 | 1 |  | 859 | 96.3 | 9.06 |
| 10800138 | histone H2B type 1-D [Homo sapiens] | 36.32 | 15.87% | 15 | 2 | 2 | 2 |  | 126 | 13.9 | 10.32 |
| 10800140 | histone H2B type 1-B [Homo sapiens] | 102.74 | 20.63% | 15 | 2 | 2 | 3 |  | 126 | 13.9 | 10.32 |
| 10800144 | histone cluster 1, H2aj [Homo sapiens] | 68.59 | 27.34% | 19 | 3 | 3 | 4 |  | 128 | 13.9 | 10.89 |
| 10835051 | cysteine--tRNA ligase, cytoplasmic isoform b [Homo sapiens] | 132.24 | 13.37% | 4 | 9 | 9 | 9 |  | 748 | 85.4 | 6.76 |
| 10835143 | complement decay-accelerating factor isoform 1 preproprotein [Homo sapiens] | 29.31 | 1.84% | 2 | 1 | 1 | 1 |  | 381 | 41.4 | 7.59 |
| 10863927 | peptidyl-prolyl cis-trans isomerase A [Homo sapiens] | 0 | 6.67% | 1 | 1 | 1 | 1 |  | 165 | 18 | 7.81 |
| 10863945 | X-ray repair cross-complementing protein 5 [Homo sapiens] | 275.84 | 25.14% | 1 | 18 | 18 | 19 |  | 732 | 82.7 | 5.81 |
| 10947139 | arginase-1 isoform 2 [Homo sapiens] | 0 | 3.42% | 2 | 1 | 1 | 1 |  | 322 | 34.7 | 7.21 |
| 11024682 | insulin-like growth factor 1 isoform 4 preproprotein [Homo sapiens] | 609.49 | 22.69% | 4 | 20 | 43 | 54 |  | 1529 | 162.1 | 7.94 |
| 11056036 | tubulin--tyrosine ligase-like protein 12 [Homo sapiens] | 95.28 | 10.87% | 1 | 5 | 5 | 5 |  | 644 | 74.4 | 5.53 |
| 11140811 | cullin-4A isoform 2 [Homo sapiens] | 0 | 1.67% | 4 | 1 | 1 | 1 |  | 659 | 76.8 | 7.18 |
| 11342676 | cdc42-interacting protein 4 [Homo sapiens] | 0 | 3.30% | 1 | 2 | 2 | 2 |  | 545 | 62.6 | 5.33 |
| 11386183 | wiskott-Aldrich syndrome protein family member 2 isoform 1 [Homo sapiens] | 39.24 | 1.61% | 1 | 1 | 1 | 1 |  | 498 | 54.3 | 5.53 |
| 11559929 | coatomer subunit gamma-1 [Homo sapiens] | 51.76 | 3.78% | 2 | 3 | 3 | 3 |  | 874 | 97.7 | 5.47 |
| 11968182 | 40S ribosomal protein S18 [Homo sapiens] | 0 | 5.92% | 1 | 1 | 1 | 1 |  | 152 | 17.7 | 10.99 |
| 12025678 | alpha-actinin-4 [Homo sapiens] | 235.65 | 33.59% | 4 | 16 | 23 | 24 |  | 911 | 104.8 | 5.44 |
| 12056468 | junction plakoglobin [Homo sapiens] | 133.61 | 17.05% | 2 | 11 | 11 | 12 |  | 745 | 81.7 | 6.14 |
| 12667788 | myosin-9 [Homo sapiens] | 0 | 0.24% | 1 | 1 | 1 | 1 |  | 2934 | 336.2 | 5.92 |
| 13435375 | glucocorticoid modulatory element-binding protein 1 isoform 2 [Homo sapiens] | 0 | 1.24% | 2 | 1 | 1 | 1 |  | 563 | 61.3 | 4.81 |
| 13518026 | LIM and senescent cell antigen-like-containing domain protein 1 isoform b [Homo sapiens] | 595.74 | 30.55% | 4 | 3 | 31 | 49 |  | 969 | 106.6 | 7.94 |
| 14043024 | BAG family molecular chaperone regulator 3 [Homo sapiens] | 73.14 | 15.30% | 1 | 9 | 9 | 9 |  | 575 | 61.6 | 6.95 |
| 14141161 | heterogeneous nuclear ribonucleoprotein U isoform b [Homo sapiens] | 31.93 | 8.06% | 2 | 5 | 5 | 5 |  | 806 | 88.9 | 5.78 |
| 14141170 | metastasis-associated protein MTA2 [Homo sapiens] | 80.45 | 13.02% | 2 | 8 | 8 | 8 |  | 668 | 75 | 9.66 |
| 14149738 | neurolysin, mitochondrial [Homo sapiens] | 37.55 | 1.42% | 1 | 1 | 1 | 1 |  | 704 | 80.6 | 6.64 |
| 14165274 | protein Hook homolog 3 [Homo sapiens] | 29.6 | 1.25% | 1 | 1 | 1 | 1 |  | 718 | 83.1 | 5.17 |
| 14210536 | tubulin beta-6 chain [Homo sapiens] | 133.07 | 14.57% | 3 | 1 | 5 | 6 |  | 446 | 49.8 | 4.88 |
| 14389309 | tubulin alpha-1C chain [Homo sapiens] | 130.29 | 23.39% | 10 | 7 | 7 | 7 |  | 449 | 49.9 | 5.1 |
| 14591904 | ATP-dependent DNA helicase Q1 [Homo sapiens] | 28.09 | 1.23% | 1 | 1 | 1 | 1 |  | 649 | 73.4 | 7.88 |
| 15100175 | 1-acyl-sn-glycerol-3-phosphate acyltransferase alpha [Homo sapiens] | 0 | 3.53% | 1 | 1 | 1 | 1 |  | 283 | 31.7 | 9.38 |
| 15149465 | caldesmon isoform 5 [Homo sapiens] | 146.24 | 15.60% | 5 | 6 | 6 | 6 |  | 532 | 61.2 | 6.71 |
| 15431301 | 60S ribosomal protein L7 [Homo sapiens] | 0 | 4.03% | 1 | 1 | 1 | 1 |  | 248 | 29.2 | 10.65 |
| 15431310 | keratin, type I cytoskeletal 14 [Homo sapiens] | 441.97 | 34.53% | 15 | 3 | 16 | 22 |  | 472 | 51.6 | 5.16 |
| 16507237 | 78 kDa glucose-regulated protein precursor [Homo sapiens] | 1437.33 | 56.88% | 3 | 37 | 39 | 63 |  | 654 | 72.3 | 5.16 |
| 16507243 | GSK-3-binding protein FRAT2 [Homo sapiens] | 0 | 3.43% | 1 | 1 | 1 | 1 |  | 233 | 24 | 5.52 |
| 16579885 | 60S ribosomal protein L4 [Homo sapiens] | 38.2 | 7.96% | 1 | 2 | 2 | 2 |  | 427 | 47.7 | 11.06 |
| 16751921 | dermcidin preproprotein [Homo sapiens] | 113.99 | 35.45% | 1 | 4 | 4 | 4 |  | 110 | 11.3 | 6.54 |
| 17933772 | protein S100-A16 [Homo sapiens] | 30.55 | 10.68% | 1 | 1 | 1 | 1 |  | 103 | 11.8 | 6.79 |
| 17986283 | tubulin alpha-1A chain [Homo sapiens] | 30.29 | 11.09% | 3 | 1 | 4 | 4 |  | 451 | 50.1 | 5.06 |
| 17998551 | serpin B12 [Homo sapiens] | 36.26 | 3.91% | 1 | 4 | 4 | 4 |  | 1100 | 122.2 | 6.19 |
| 17999541 | vacuolar protein sorting-associated protein 35 [Homo sapiens] | 208.41 | 21.36% | 1 | 14 | 14 | 14 |  | 796 | 91.6 | 5.49 |
| 18105032 | collagen alpha-1(X) chain precursor [Homo sapiens] | 138.99 | 15.40% | 10 | 4 | 13 | 19 |  | 1143 | 118.1 | 8.27 |
| 18201905 | glucose-6-phosphate isomerase isoform 2 [Homo sapiens] | 0 | 3.05% | 2 | 1 | 1 | 1 |  | 558 | 63.1 | 8.32 |
| 18390331 | elongation factor G, mitochondrial [Homo sapiens] | 55.98 | 4.79% | 1 | 4 | 4 | 4 |  | 751 | 83.4 | 7.01 |
| 18426904 | ATPase WRNIP1 isoform 2 [Homo sapiens] | 23.48 | 3.59% | 2 | 2 | 2 | 2 |  | 640 | 69.4 | 6.27 |
| 18860831 | dynamin-like 120 kDa protein, mitochondrial isoform 2 [Homo sapiens] | 37.8 | 2.06% | 8 | 2 | 2 | 2 |  | 924 | 107.5 | 7.99 |
| 18860916 | 5'-3' exoribonuclease 2 [Homo sapiens] | 0 | 1.26% | 1 | 1 | 1 | 1 |  | 950 | 108.5 | 7.47 |
| 19743823 | integrin beta-1 isoform 1A precursor [Homo sapiens] | 106.77 | 11.28% | 2 | 7 | 7 | 7 |  | 798 | 88.4 | 5.39 |
| 19923096 | cytokine receptor-like factor 2 isoform 1 precursor [Homo sapiens] | 117.19 | 9.42% | 1 | 10 | 10 | 11 |  | 1168 | 128.5 | 7.42 |
| 19923142 | importin subunit beta-1 isoform 1 [Homo sapiens] | 51.62 | 3.08% | 2 | 2 | 2 | 2 |  | 876 | 97.1 | 4.78 |
| 19923981 | IQ domain-containing protein D [Homo sapiens] | 142.58 | 2.08% | 5 | 1 | 2 | 7 |  | 866 | 98.8 | 7.12 |
| 20070125 | protein disulfide-isomerase precursor [Homo sapiens] | 0 | 0.58% | 1 | 1 | 1 | 1 |  | 1378 | 153.3 | 5.87 |
| 20070344 | threonine--tRNA ligase, mitochondrial isoform a [Homo sapiens] | 45.08 | 1.39% | 1 | 1 | 1 | 1 |  | 718 | 81 | 7.3 |
| 20127408 | trifunctional enzyme subunit alpha, mitochondrial precursor [Homo sapiens] | 316.62 | 28.18% | 1 | 18 | 18 | 21 |  | 763 | 82.9 | 9.04 |
| 20127499 | serine/arginine-rich splicing factor 6 [Homo sapiens] | 62.07 | 5.52% | 2 | 2 | 2 | 2 |  | 344 | 39.6 | 11.43 |
| 20149547 | ribosomal protein S6 kinase alpha-1 isoform a [Homo sapiens] | 84.18 | 9.12% | 18 | 2 | 7 | 7 |  | 735 | 82.7 | 7.83 |
| 20149594 | heat shock protein HSP 90-beta [Homo sapiens] | 61.97 | 13.84% | 2 | 7 | 14 | 14 |  | 1091 | 125.4 | 5.44 |
| 20270303 | mitochondrial Rho GTPase 2 [Homo sapiens] | 40.41 | 1.62% | 1 | 1 | 1 | 1 |  | 618 | 68.1 | 5.86 |
| 20357552 | src substrate cortactin isoform a [Homo sapiens] | 424.83 | 41.45% | 3 | 25 | 25 | 25 |  | 550 | 61.5 | 5.4 |
| 21361657 | protein disulfide-isomerase A3 precursor [Homo sapiens] | 0 | 1.58% | 1 | 1 | 1 | 1 |  | 505 | 56.7 | 6.35 |
| 21489959 | immunoglobulin J chain precursor [Homo sapiens] | 0 | 1.68% | 1 | 2 | 2 | 2 |  | 1131 | 126.3 | 5.11 |
| 21536286 | creatine kinase B-type [Homo sapiens] | 0 | 3.41% | 1 | 1 | 1 | 1 |  | 381 | 42.6 | 5.59 |
| 21536288 | creatine kinase M-type [Homo sapiens] | 30.45 | 1.84% | 1 | 1 | 1 | 1 |  | 381 | 43.1 | 7.25 |
| 21536320 | heterogeneous nuclear ribonucleoprotein U-like protein 1 isoform d [Homo sapiens] | 37.23 | 3.04% | 2 | 2 | 2 | 2 |  | 756 | 84.7 | 8.78 |
| 21536413 | myc box-dependent-interacting protein 1 isoform 7 [Homo sapiens] | 41.53 | 2.18% | 1 | 2 | 2 | 2 |  | 1101 | 119.7 | 5.26 |
| 21614544 | protein S100-A8 [Homo sapiens] | 49.82 | 40.86% | 1 | 5 | 5 | 5 |  | 93 | 10.8 | 7.03 |
| 21914927 | lymphoid-specific helicase [Homo sapiens] | 0 | 1.19% | 1 | 1 | 1 | 1 |  | 838 | 97 | 7.93 |
| 22027538 | programmed cell death 6-interacting protein isoform 1 [Homo sapiens] | 99.23 | 12.79% | 3 | 11 | 11 | 11 |  | 868 | 96 | 6.52 |
| 23110925 | proteasome subunit beta type-6 [Homo sapiens] | 0 | 2.53% | 1 | 1 | 1 | 1 |  | 435 | 48.6 | 5.85 |
| 23308689 | RNA pseudouridylate synthase domain-containing protein 2 [Homo sapiens] | 30.13 | 2.20% | 1 | 1 | 1 | 1 |  | 545 | 61.3 | 7.17 |
| 23503325 | mirror-image polydactyly gene 1 protein [Homo sapiens] | 73.09 | 9.13% | 1 | 8 | 9 | 9 |  | 1041 | 117.5 | 5.78 |
| 23510340 | ubiquitin-like modifier-activating enzyme 1 [Homo sapiens] | 339.18 | 26.65% | 1 | 21 | 21 | 24 |  | 1058 | 117.8 | 5.76 |
| 23510448 | DNA replication licensing factor MCM5 [Homo sapiens] | 38.9 | 1.77% | 1 | 1 | 1 | 1 |  | 734 | 82.2 | 8.37 |
| 23510451 | acylamino-acid-releasing enzyme [Homo sapiens] | 36.47 | 6.01% | 1 | 4 | 4 | 4 |  | 732 | 81.2 | 5.48 |
| 24234686 | heat shock cognate 71 kDa protein isoform 2 [Homo sapiens] | 35.83 | 5.27% | 8 | 2 | 2 | 2 |  | 493 | 53.5 | 5.86 |
| 24234688 | stress-70 protein, mitochondrial precursor [Homo sapiens] | 169.51 | 12.81% | 1 | 7 | 7 | 8 |  | 679 | 73.6 | 6.16 |
| 24234690 | double-strand break repair protein MRE11A isoform 2 [Homo sapiens] | 44.34 | 6.62% | 2 | 4 | 4 | 4 |  | 680 | 77.6 | 5.82 |
| 24234756 | interleukin enhancer-binding factor 3 isoform c [Homo sapiens] | 148.56 | 17.54% | 5 | 8 | 10 | 10 |  | 690 | 74.6 | 8.24 |
| 24308039 | E3 UFM1-protein ligase 1 [Homo sapiens] | 55.23 | 3.78% | 1 | 3 | 3 | 3 |  | 794 | 89.5 | 6.79 |
| 24308113 | KIF1-binding protein [Homo sapiens] | 31.82 | 3.86% | 1 | 2 | 2 | 2 |  | 621 | 71.8 | 5.49 |
| 24308179 | CTTNBP2 N-terminal-like protein [Homo sapiens] | 40.85 | 2.50% | 1 | 2 | 2 | 2 |  | 639 | 70.1 | 8.06 |
| 24430192 | keratin, type I cytoskeletal 16 [Homo sapiens] | 445.49 | 28.96% | 15 | 4 | 15 | 20 |  | 473 | 51.2 | 5.05 |
| 24497453 | nuclear pore complex protein Nup88 [Homo sapiens] | 35 | 2.97% | 1 | 2 | 2 | 2 |  | 741 | 83.5 | 5.69 |
| 24638433 | nicastrin precursor [Homo sapiens] | 0 | 1.55% | 1 | 1 | 1 | 1 |  | 709 | 78.4 | 5.99 |
| 24797086 | importin-5 [Homo sapiens] | 69.84 | 6.82% | 3 | 6 | 6 | 6 |  | 1115 | 125.5 | 4.92 |
| 25121987 | condensin complex subunit 2 [Homo sapiens] | 33.45 | 1.48% | 1 | 1 | 1 | 1 |  | 741 | 82.5 | 5.06 |
| 25306283 | ribosome-releasing factor 2, mitochondrial isoform 3 [Homo sapiens] | 0 | 2.34% | 3 | 1 | 1 | 1 |  | 513 | 57 | 7.09 |
| 25777602 | 26S proteasome non-ATPase regulatory subunit 2 [Homo sapiens] | 0 | 0.89% | 1 | 1 | 1 | 1 |  | 1350 | 151.6 | 6.02 |
| 25777612 | 26S proteasome non-ATPase regulatory subunit 3 [Homo sapiens] | 0 | 4.87% | 1 | 2 | 2 | 2 |  | 534 | 60.9 | 8.44 |
| 27436946 | lamin isoform A [Homo sapiens] | 1046.73 | 55.87% | 7 | 37 | 37 | 44 |  | 664 | 74.1 | 7.02 |
| 27477041 | AP-2 complex subunit alpha-2 isoform 2 [Homo sapiens] | 23.59 | 2.88% | 4 | 2 | 2 | 2 |  | 939 | 103.9 | 6.96 |
| 27477138 | zinc finger CCCH-type antiviral protein 1 isoform 2 [Homo sapiens] | 78.39 | 2.15% | 2 | 1 | 1 | 1 |  | 699 | 77.9 | 8.38 |
| 27735067 | uncharacterized protein C19orf21 [Homo sapiens] | 0 | 4.42% | 1 | 2 | 2 | 2 |  | 679 | 75.3 | 6.83 |
| 27777657 | serpin A12 precursor [Homo sapiens] | 61.08 | 3.16% | 3 | 1 | 4 | 5 |  | 1014 | 113.6 | 8.38 |
| 27881506 | ATP-binding cassette sub-family F member 2 isoform a [Homo sapiens] | 28.09 | 8.35% | 2 | 5 | 5 | 5 |  | 623 | 71.2 | 7.37 |
| 28076869 | serpin B4 [Homo sapiens] | 21.32 | 8.46% | 2 | 3 | 3 | 3 |  | 390 | 44.8 | 6.21 |
| 28302131 | hemoglobin subunit gamma-1 [Homo sapiens] | 48.66 | 6.80% | 5 | 1 | 1 | 1 |  | 147 | 16.1 | 7.2 |
| 28460688 | alpha-taxilin [Homo sapiens] | 103.18 | 10.26% | 1 | 5 | 5 | 5 |  | 546 | 61.9 | 6.52 |
| 28558975 | mediator of RNA polymerase II transcription subunit 17 [Homo sapiens] | 0 | 1.69% | 1 | 1 | 1 | 1 |  | 651 | 72.8 | 7.44 |
| 28631173 | molybdopterin synthase catalytic subunit small subunit MOCS2A [Homo sapiens] | 129.8 | 16.53% | 1 | 16 | 16 | 16 |  | 1113 | 121.9 | 6.7 |
| 29029559 | exportin-2 isoform 1 [Homo sapiens] | 63.97 | 6.59% | 2 | 10 | 10 | 10 |  | 1579 | 176.9 | 6.33 |
| 29788785 | tubulin beta chain [Homo sapiens] | 158.79 | 22.30% | 9 | 4 | 8 | 11 |  | 444 | 49.6 | 4.89 |
| 29826282 | protein phosphatase 1G [Homo sapiens] | 85.11 | 10.44% | 1 | 5 | 5 | 5 |  | 546 | 59.2 | 4.36 |
| 31377697 | procollagen galactosyltransferase 1 precursor [Homo sapiens] | 24.92 | 5.14% | 2 | 3 | 3 | 3 |  | 622 | 71.6 | 7.31 |
| 31377806 | polymeric immunoglobulin receptor precursor [Homo sapiens] | 67.45 | 5.63% | 1 | 6 | 6 | 6 |  | 764 | 83.2 | 5.74 |
| 31541941 | heat shock 70 kDa protein 4L [Homo sapiens] | 59.45 | 5.96% | 1 | 2 | 4 | 4 |  | 839 | 94.5 | 5.88 |
| 31543397 | phosphoglycerate kinase 2 [Homo sapiens] | 0 | 3.60% | 2 | 1 | 1 | 1 |  | 417 | 44.8 | 8.54 |
| 31543667 | ATP-dependent RNA helicase SUPV3L1, mitochondrial precursor [Homo sapiens] | 41.93 | 1.65% | 1 | 1 | 1 | 1 |  | 786 | 87.9 | 7.99 |
| 32189394 | ATP synthase subunit beta, mitochondrial precursor [Homo sapiens] | 34.88 | 4.91% | 1 | 2 | 2 | 2 |  | 529 | 56.5 | 5.4 |
| 32307144 | procollagen-lysine,2-oxoglutarate 5-dioxygenase 1 precursor [Homo sapiens] | 155.37 | 19.12% | 3 | 12 | 12 | 12 |  | 727 | 83.5 | 6.95 |
| 32455266 | peroxiredoxin-1 [Homo sapiens] | 0 | 4.02% | 3 | 1 | 1 | 1 |  | 199 | 22.1 | 8.13 |
| 32483412 | gap junction alpha-5 protein [Homo sapiens] | 45.63 | 0.60% | 1 | 1 | 1 | 1 |  | 1506 | 168.5 | 7.72 |
| 32698730 | nuclear fragile X mental retardation-interacting protein 2 [Homo sapiens] | 34.07 | 4.89% | 1 | 3 | 3 | 3 |  | 695 | 76.1 | 8.7 |
| 33239445 | eukaryotic translation initiation factor 3 subunit B [Homo sapiens] | 0 | 2.70% | 1 | 2 | 2 | 2 |  | 814 | 92.4 | 5 |
| 33286418 | pyruvate kinase isozymes M1/M2 isoform a [Homo sapiens] | 288.62 | 34.09% | 8 | 13 | 13 | 13 |  | 531 | 57.9 | 7.84 |
| 33286446 | opioid growth factor receptor [Homo sapiens] | 0 | 1.18% | 1 | 1 | 1 | 1 |  | 677 | 73.3 | 4.84 |
| 33469919 | DNA replication licensing factor MCM4 [Homo sapiens] | 72.98 | 8.46% | 1 | 6 | 6 | 6 |  | 863 | 96.5 | 6.74 |
| 33469964 | SURP and G-patch domain-containing protein 1 [Homo sapiens] | 0 | 1.71% | 1 | 1 | 1 | 1 |  | 645 | 72.4 | 7.61 |
| 33469968 | DNA replication licensing factor MCM7 isoform 1 [Homo sapiens] | 365.07 | 33.94% | 2 | 19 | 19 | 22 |  | 719 | 81.3 | 6.46 |
| 34452173 | integrin alpha-X precursor [Homo sapiens] | 477.45 | 16.18% | 5 | 3 | 31 | 41 |  | 1805 | 196.8 | 6.79 |
| 38016947 | complement C5 preproprotein [Homo sapiens] | 17.02 | 0.66% | 1 | 1 | 1 | 1 |  | 1676 | 188.2 | 6.52 |
| 38195082 | von Willebrand factor A domain-containing protein 5A isoform 2 [Homo sapiens] | 0 | 6.27% | 2 | 2 | 2 | 2 |  | 415 | 45.9 | 5.16 |
| 38201710 | probable ATP-dependent RNA helicase DDX17 isoform 1 [Homo sapiens] | 213.83 | 26.75% | 3 | 15 | 16 | 17 |  | 729 | 80.2 | 8.27 |
| 38202257 | neutral alpha-glucosidase AB isoform 2 precursor [Homo sapiens] | 151.64 | 13.77% | 2 | 9 | 9 | 9 |  | 944 | 106.8 | 6.14 |
| 38327039 | heat shock 70 kDa protein 4 [Homo sapiens] | 103.26 | 15.48% | 1 | 9 | 10 | 10 |  | 840 | 94.3 | 5.19 |
| 38492358 | pentatricopeptide repeat-containing protein 1 [Homo sapiens] | 0 | 1.57% | 2 | 1 | 1 | 1 |  | 700 | 78.8 | 8.59 |
| 38569423 | ATP-citrate synthase isoform 2 [Homo sapiens] | 94.99 | 11.27% | 2 | 10 | 10 | 10 |  | 1091 | 119.7 | 7.33 |
| 38683855 | pentatricopeptide repeat domain-containing protein 3, mitochondrial precursor [Homo sapiens] | 0 | 1.31% | 1 | 1 | 1 | 1 |  | 689 | 78.5 | 6.42 |
| 39777604 | rho guanine nucleotide exchange factor 1 isoform 3 [Homo sapiens] | 0 | 1.02% | 3 | 1 | 1 | 1 |  | 879 | 98.7 | 5.57 |
| 39780552 | protein VAC14 homolog [Homo sapiens] | 0 | 2.05% | 1 | 2 | 2 | 2 |  | 782 | 87.9 | 6.13 |
| 39780588 | pre-rRNA-processing protein TSR1 homolog [Homo sapiens] | 0 | 1.12% | 1 | 1 | 1 | 1 |  | 804 | 91.8 | 7.42 |
| 39930375 | protein enabled homolog isoform b [Homo sapiens] | 163.87 | 15.61% | 2 | 9 | 9 | 9 |  | 570 | 63.9 | 6.43 |
| 39995082 | tRNA (cytosine(34)-C(5))-methyltransferase isoform 1 [Homo sapiens] | 90.11 | 17.73% | 2 | 10 | 10 | 10 |  | 767 | 86.4 | 6.77 |
| 41152097 | A-kinase anchor protein 17A isoform 1 [Homo sapiens] | 0 | 1.29% | 1 | 1 | 1 | 1 |  | 695 | 80.7 | 9.73 |
| 41281996 | HEAT repeat-containing protein 3 [Homo sapiens] | 31.19 | 1.91% | 1 | 1 | 1 | 1 |  | 680 | 74.5 | 5.11 |
| 41350201 | epsin-1 isoform c [Homo sapiens] | 24.88 | 2.18% | 4 | 1 | 1 | 1 |  | 550 | 57.5 | 4.88 |
| 41350320 | melanoma-associated antigen D2 [Homo sapiens] | 28.48 | 4.79% | 1 | 3 | 3 | 3 |  | 606 | 64.9 | 9.32 |
| 41399285 | 60 kDa heat shock protein, mitochondrial [Homo sapiens] | 29.63 | 5.06% | 1 | 3 | 3 | 3 |  | 573 | 61 | 5.87 |
| 41406069 | histone H2A.V isoform 4 [Homo sapiens] | 0 | 13.64% | 18 | 1 | 1 | 1 |  | 66 | 6.9 | 11.06 |
| 42518068 | trans-Golgi network integral membrane protein 2 isoform 1 precursor [Homo sapiens] | 122.9 | 16.70% | 4 | 6 | 6 | 6 |  | 437 | 45.9 | 5.63 |
| 42544123 | splicing factor 1 isoform 3 [Homo sapiens] | 47.49 | 10.40% | 6 | 4 | 4 | 6 |  | 548 | 59.7 | 9.5 |
| 42544159 | heat shock protein 105 kDa [Homo sapiens] | 100.27 | 13.05% | 1 | 8 | 9 | 9 |  | 858 | 96.8 | 5.39 |
| 42734503 | caprin-1 isoform 2 [Homo sapiens] | 124.7 | 10.52% | 2 | 7 | 7 | 7 |  | 694 | 76.8 | 5.12 |
| 44890059 | involucrin [Homo sapiens] | 0 | 1.71% | 1 | 1 | 1 | 1 |  | 585 | 68.4 | 4.61 |
| 44921615 | exocyst complex component 8 [Homo sapiens] | 0 | 1.24% | 1 | 1 | 1 | 1 |  | 725 | 81.7 | 5.49 |
| 45243501 | bcl-2-like protein 13 isoform a [Homo sapiens] | 29.24 | 9.48% | 9 | 4 | 4 | 4 |  | 485 | 52.7 | 4.44 |
| 45439306 | aspartate--tRNA ligase, cytoplasmic [Homo sapiens] | 36.92 | 8.38% | 1 | 4 | 4 | 4 |  | 501 | 57.1 | 6.55 |
| 45439327 | periplakin [Homo sapiens] | 33.18 | 0.46% | 1 | 1 | 1 | 1 |  | 1756 | 204.6 | 5.58 |
| 46358428 | intraflagellar transport protein 172 homolog [Homo sapiens] | 27.36 | 0.40% | 1 | 1 | 1 | 1 |  | 1749 | 197.5 | 6.13 |
| 46367787 | polyadenylate-binding protein 1 [Homo sapiens] | 435.58 | 39.31% | 4 | 15 | 23 | 26 |  | 636 | 70.6 | 9.5 |
| 46488921 | astrotactin-1 isoform 2 precursor [Homo sapiens] | 0 | 1.32% | 2 | 1 | 1 | 1 |  | 1216 | 135 | 5.15 |
| 46592991 | GRIP1-associated protein 1 isoform 1 [Homo sapiens] | 0 | 1.19% | 1 | 1 | 1 | 1 |  | 841 | 95.9 | 5.11 |
| 46593000 | GRIP1-associated protein 1 isoform 2 [Homo sapiens] | 0 | 1.44% | 2 | 1 | 1 | 1 |  | 625 | 71.8 | 5.38 |
| 47132589 | serine/threonine-protein kinase N1 isoform 2 [Homo sapiens] | 0 | 0.96% | 2 | 1 | 1 | 1 |  | 942 | 103.9 | 6.37 |
| 47132595 | phosphate carrier protein, mitochondrial isoform b precursor [Homo sapiens] | 0 | 1.94% | 2 | 1 | 1 | 1 |  | 361 | 39.9 | 9.36 |
| 47132620 | keratin, type II cytoskeletal 2 epidermal [Homo sapiens] | 1212.48 | 65.41% | 7 | 32 | 42 | 54 |  | 639 | 65.4 | 8 |
| 47271406 | 7SK snRNA methylphosphate capping enzyme isoform A [Homo sapiens] | 27.64 | 3.63% | 2 | 3 | 3 | 3 |  | 689 | 74.3 | 9.57 |
| 47458820 | signal transducer and activator of transcription 3 isoform 3 [Homo sapiens] | 126.33 | 12.47% | 3 | 6 | 6 | 6 |  | 722 | 83.1 | 7.12 |
| 47825361 | non-specific cytotoxic cell receptor protein 1 homolog [Homo sapiens] | 0 | 4.00% | 1 | 1 | 1 | 1 |  | 275 | 30.8 | 6.62 |
| 48255891 | glucosidase 2 subunit beta isoform 2 precursor [Homo sapiens] | 200.51 | 13.33% | 2 | 7 | 7 | 11 |  | 525 | 59.1 | 4.42 |
| 50053795 | eukaryotic translation initiation factor 4B [Homo sapiens] | 309.07 | 29.79% | 1 | 17 | 17 | 19 |  | 611 | 69.1 | 5.73 |
| 50511939 | exosome complex component RRP40 isoform 2 [Homo sapiens] | 0 | 4.88% | 2 | 1 | 1 | 1 |  | 164 | 17.2 | 9.28 |
| 50727002 | pseudouridylate synthase 7 homolog [Homo sapiens] | 31.56 | 4.84% | 1 | 3 | 3 | 3 |  | 661 | 75 | 6.37 |
| 53729344 | plakophilin-1 isoform 1a [Homo sapiens] | 66.25 | 6.61% | 2 | 4 | 4 | 4 |  | 726 | 80.4 | 8.97 |
| 53759107 | argininosuccinate synthase [Homo sapiens] | 0 | 1.94% | 1 | 1 | 1 | 1 |  | 412 | 46.5 | 8.02 |
| 54607135 | mitochondrial import receptor subunit TOM70 [Homo sapiens] | 0 | 1.32% | 1 | 1 | 1 | 1 |  | 608 | 67.4 | 7.12 |
| 54792067 | small ubiquitin-related modifier 1 isoform b precursor [Homo sapiens] | 0 | 9.21% | 2 | 1 | 1 | 1 |  | 76 | 8.8 | 5.73 |
| 54792092 | ATM interactor [Homo sapiens] | 29.48 | 0.85% | 1 | 1 | 1 | 1 |  | 823 | 88.3 | 5.16 |
| 54860105 | rho GTPase-activating protein 17 isoform 2 [Homo sapiens] | 0 | 1.12% | 2 | 1 | 1 | 1 |  | 803 | 87.6 | 7.59 |
| 55770844 | catenin alpha-1 [Homo sapiens] | 0 | 1.21% | 1 | 1 | 1 | 1 |  | 906 | 100 | 6.29 |
| 55953087 | nucleolar GTP-binding protein 1 [Homo sapiens] | 54.87 | 3.63% | 1 | 2 | 2 | 2 |  | 634 | 73.9 | 9.5 |
| 55956788 | nucleolin [Homo sapiens] | 187.45 | 25.07% | 1 | 15 | 15 | 15 |  | 710 | 76.6 | 4.7 |
| 55956899 | keratin, type I cytoskeletal 9 [Homo sapiens] | 821.25 | 50.24% | 1 | 24 | 25 | 33 |  | 623 | 62 | 5.24 |
| 56549117 | dynamin-1 isoform 2 [Homo sapiens] | 24.73 | 2.35% | 9 | 2 | 2 | 2 |  | 851 | 96 | 7.01 |
| 56549119 | dynamin-2 isoform 3 [Homo sapiens] | 0 | 2.54% | 9 | 2 | 2 | 2 |  | 866 | 97.6 | 7.64 |
| 56676330 | heterochromatin protein 1-binding protein 3 [Homo sapiens] | 78.44 | 7.96% | 1 | 4 | 4 | 4 |  | 553 | 61.2 | 9.67 |
| 57013276 | tubulin alpha-1B chain [Homo sapiens] | 37.38 | 11.09% | 5 | 1 | 4 | 4 |  | 451 | 50.1 | 5.06 |
| 57165424 | cullin-4A isoform 1 [Homo sapiens] | 99.11 | 9.88% | 3 | 3 | 7 | 7 |  | 759 | 87.6 | 8.13 |
| 57863257 | T-complex protein 1 subunit alpha isoform a [Homo sapiens] | 62.27 | 9.35% | 2 | 4 | 4 | 4 |  | 556 | 60.3 | 6.11 |
| 57864582 | hornerin [Homo sapiens] | 215.16 | 9.96% | 2 | 8 | 8 | 8 |  | 2850 | 282.2 | 10.04 |
| 58530840 | desmoplakin isoform I [Homo sapiens] | 199.75 | 8.74% | 2 | 23 | 23 | 23 |  | 2871 | 331.6 | 6.81 |
| 58530845 | zyxin [Homo sapiens] | 95.97 | 10.49% | 1 | 4 | 4 | 4 |  | 572 | 61.2 | 6.67 |
| 60097902 | filaggrin [Homo sapiens] | 42.17 | 2.61% | 1 | 2 | 2 | 2 |  | 4061 | 434.9 | 9.25 |
| 61743954 | neuroblast differentiation-associated protein AHNAK isoform 1 [Homo sapiens] | 39.83 | 3.82% | 1 | 2 | 2 | 3 |  | 5890 | 628.7 | 6.15 |
| 61744481 | 4F2 cell-surface antigen heavy chain isoform e [Homo sapiens] | 74.72 | 29.58% | 4 | 13 | 13 | 13 |  | 568 | 61.8 | 5.21 |
| 61744483 | 4F2 cell-surface antigen heavy chain isoform f [Homo sapiens] | 424.08 | 31.19% | 4 | 15 | 15 | 20 |  | 529 | 57.9 | 5.35 |
| 61835172 | fragile X mental retardation syndrome-related protein 1 isoform c [Homo sapiens] | 38.38 | 1.49% | 4 | 1 | 1 | 1 |  | 536 | 59.9 | 6.77 |
| 61888896 | roundabout homolog 2 isoform ROBO2b precursor [Homo sapiens] | 500.61 | 10.94% | 8 | 10 | 20 | 30 |  | 1929 | 211.4 | 5.72 |
| 62122917 | filaggrin-2 [Homo sapiens] | 26.12 | 3.93% | 1 | 3 | 3 | 3 |  | 2391 | 247.9 | 8.31 |
| 62243374 | mitotic spindle assembly checkpoint protein MAD1 [Homo sapiens] | 39.87 | 3.76% | 1 | 3 | 3 | 3 |  | 718 | 83 | 5.92 |
| 62739166 | procollagen-lysine,2-oxoglutarate 5-dioxygenase 2 isoform 2 precursor [Homo sapiens] | 38.53 | 1.63% | 2 | 1 | 1 | 1 |  | 737 | 84.6 | 6.71 |
| 62750354 | matrin-3 isoform a [Homo sapiens] | 25.21 | 1.65% | 2 | 1 | 1 | 1 |  | 847 | 94.6 | 6.25 |
| 62912457 | delta-1-pyrroline-5-carboxylate synthase isoform 2 [Homo sapiens] | 167.94 | 11.73% | 2 | 8 | 8 | 9 |  | 793 | 87 | 7.12 |
| 63252904 | tropomyosin alpha-1 chain isoform 6 [Homo sapiens] | 0 | 6.94% | 16 | 2 | 2 | 2 |  | 245 | 28.4 | 4.77 |
| 66346685 | plasminogen activator inhibitor 1 RNA-binding protein isoform 4 [Homo sapiens] | 67.12 | 4.13% | 4 | 1 | 1 | 1 |  | 387 | 42.4 | 8.44 |
| 66932947 | alpha-2-macroglobulin precursor [Homo sapiens] | 0 | 0.47% | 1 | 1 | 1 | 1 |  | 1474 | 163.2 | 6.42 |
| 66933005 | calnexin precursor [Homo sapiens] | 183.23 | 15.20% | 1 | 9 | 9 | 10 |  | 592 | 67.5 | 4.6 |
| 67189747 | 60S ribosomal protein L6 [Homo sapiens] | 37.25 | 2.78% | 1 | 1 | 1 | 1 |  | 288 | 32.7 | 10.58 |
| 67191208 | polyubiquitin-C [Homo sapiens] | 41.81 | 32.85% | 4 | 2 | 2 | 2 |  | 685 | 77 | 7.66 |
| 67782365 | keratin, type II cytoskeletal 7 [Homo sapiens] | 180.53 | 12.58% | 14 | 3 | 7 | 7 |  | 469 | 51.4 | 5.48 |
| 68077166 | dnaJ homolog subfamily C member 21 isoform 2 [Homo sapiens] | 60.33 | 3.01% | 2 | 1 | 1 | 1 |  | 531 | 62 | 5.47 |
| 68509926 | putative pre-mRNA-splicing factor ATP-dependent RNA helicase DHX15 [Homo sapiens] | 31.25 | 1.38% | 1 | 1 | 1 | 1 |  | 795 | 90.9 | 7.46 |
| 68509932 | myelin basic protein isoform 4 [Homo sapiens] | 69.92 | 22.50% | 6 | 3 | 3 | 5 |  | 160 | 17.3 | 11.14 |
| 68563515 | keratinocyte proline-rich protein [Homo sapiens] | 44.89 | 1.55% | 1 | 1 | 1 | 1 |  | 579 | 64.1 | 8.27 |
| 69354671 | ATP-binding cassette sub-family F member 1 isoform a [Homo sapiens] | 0 | 3.91% | 2 | 3 | 3 | 3 |  | 845 | 95.9 | 6.8 |
| 71274107 | cell surface glycoprotein MUC18 precursor [Homo sapiens] | 0 | 1.86% | 1 | 1 | 1 | 1 |  | 646 | 71.6 | 5.76 |
| 71772942 | AP-1 complex subunit gamma-1 isoform b [Homo sapiens] | 0 | 1.34% | 2 | 1 | 1 | 1 |  | 822 | 91.3 | 6.8 |
| 74271837 | glutamine synthetase [Homo sapiens] | 0 | 5.36% | 1 | 1 | 1 | 1 |  | 373 | 42 | 6.89 |
| 75709187 | cleavage stimulation factor subunit 3 isoform 2 [Homo sapiens] | 0 | 9.71% | 2 | 1 | 1 | 1 |  | 103 | 12.1 | 7.12 |
| 76496472 | 60S ribosomal protein L3 isoform b [Homo sapiens] | 40.5 | 3.39% | 2 | 1 | 1 | 1 |  | 354 | 40.1 | 10.23 |
| 77404397 | staphylococcal nuclease domain-containing protein 1 [Homo sapiens] | 60.04 | 6.04% | 1 | 5 | 5 | 5 |  | 910 | 101.9 | 7.17 |
| 79750824 | niban-like protein 1 isoform 2 [Homo sapiens] | 145.21 | 12.01% | 2 | 8 | 8 | 10 |  | 733 | 82.6 | 6.15 |
| 83267879 | translation initiation factor eIF-2B subunit epsilon [Homo sapiens] | 28.47 | 1.11% | 1 | 1 | 1 | 1 |  | 721 | 80.3 | 5.08 |
| 83641870 | nucleophosmin isoform 3 [Homo sapiens] | 0 | 3.47% | 3 | 1 | 1 | 1 |  | 259 | 28.4 | 4.72 |
| 85067501 | arachidonate 15-lipoxygenase B isoform d [Homo sapiens] | 148.44 | 8.93% | 1 | 12 | 12 | 13 |  | 1725 | 191.7 | 5.71 |
| 88900507 | protein arginine N-methyltransferase 5 isoform b [Homo sapiens] | 37.77 | 2.90% | 2 | 2 | 2 | 2 |  | 620 | 71.3 | 6.46 |
| 88999583 | myosin light polypeptide 6 isoform 2 [Homo sapiens] | 0 | 17.88% | 1 | 1 | 1 | 1 |  | 151 | 17 | 4.55 |
| 89179321 | protein unc-45 homolog A isoform 3 [Homo sapiens] | 0 | 1.72% | 2 | 1 | 1 | 1 |  | 929 | 101.6 | 6.06 |
| 89357932 | keratin, type II cytoskeletal 78 [Homo sapiens] | 79.72 | 8.27% | 10 | 3 | 5 | 5 |  | 520 | 56.8 | 6.02 |
| 94721336 | RUN and FYVE domain-containing protein 1 isoform a [Homo sapiens] | 43.73 | 4.80% | 5 | 3 | 3 | 3 |  | 708 | 79.8 | 5.74 |
| 98986453 | myosin-3 [Homo sapiens] | 61.03 | 3.30% | 3 | 1 | 5 | 5 |  | 2151 | 247.9 | 5.85 |
| 100816392 | far upstream element-binding protein 3 [Homo sapiens] | 48.09 | 1.40% | 3 | 1 | 1 | 1 |  | 572 | 61.6 | 8.38 |
| 100913206 | ATP-dependent RNA helicase A [Homo sapiens] | 0 | 0.79% | 1 | 1 | 1 | 1 |  | 1270 | 140.9 | 6.84 |
| 105990539 | neurofilament light polypeptide [Homo sapiens] | 0 | 3.13% | 5 | 2 | 2 | 2 |  | 543 | 61.5 | 4.65 |
| 106879206 | bifunctional lysine-specific demethylase and histidyl-hydroxylase NO66 [Homo sapiens] | 31.57 | 1.56% | 1 | 1 | 1 | 1 |  | 641 | 71 | 6.46 |
| 109148542 | alanine--tRNA ligase, cytoplasmic [Homo sapiens] | 31.86 | 1.24% | 1 | 1 | 1 | 1 |  | 968 | 106.7 | 5.53 |
| 109255251 | loricrin [Homo sapiens] | 0 | 2.56% | 1 | 1 | 1 | 1 |  | 312 | 25.7 | 8.09 |
| 109637759 | calpastatin isoform f [Homo sapiens] | 44.06 | 6.00% | 3 | 3 | 3 | 3 |  | 750 | 80.2 | 5.15 |
| 109809741 | la-related protein 7 isoform 1 [Homo sapiens] | 63.65 | 4.12% | 2 | 2 | 2 | 2 |  | 582 | 66.9 | 9.55 |
| 109948279 | galectin-7 [Homo sapiens] | 0 | 20.59% | 1 | 2 | 2 | 2 |  | 136 | 15.1 | 7.62 |
| 110815838 | dysbindin domain-containing protein 1 isoform 1 [Homo sapiens] | 0 | 1.73% | 4 | 1 | 1 | 1 |  | 578 | 66.5 | 5.49 |
| 112380628 | lysosome-associated membrane glycoprotein 1 precursor [Homo sapiens] | 110.98 | 9.83% | 1 | 4 | 4 | 4 |  | 417 | 44.9 | 8.75 |
| 115298657 | protein S100-A7 [Homo sapiens] | 0 | 10.89% | 2 | 1 | 1 | 1 |  | 101 | 11.5 | 6.77 |
| 115298678 | complement C3 precursor [Homo sapiens] | 2676.22 | 40.83% | 2 | 63 | 63 | 126 |  | 1663 | 187 | 6.4 |
| 115527082 | myosin-1 [Homo sapiens] | 61.16 | 4.90% | 5 | 1 | 8 | 8 |  | 1939 | 223 | 5.74 |
| 116063573 | filamin-A isoform 1 [Homo sapiens] | 64.79 | 1.55% | 8 | 3 | 3 | 3 |  | 2639 | 279.8 | 6.05 |
| 116805340 | glycine--tRNA ligase precursor [Homo sapiens] | 157.77 | 27.60% | 1 | 17 | 17 | 17 |  | 739 | 83.1 | 7.03 |
| 117938251 | bcl-2-associated transcription factor 1 isoform 2 [Homo sapiens] | 52.9 | 4.60% | 3 | 4 | 4 | 4 |  | 869 | 100.2 | 9.95 |
| 118601081 | heterogeneous nuclear ribonucleoprotein U-like protein 2 [Homo sapiens] | 0 | 1.87% | 1 | 1 | 1 | 1 |  | 747 | 85.1 | 4.91 |
| 119372298 | pepsin A preproprotein [Homo sapiens] | 0 | 2.06% | 3 | 1 | 1 | 1 |  | 388 | 41.9 | 4.41 |
| 119395750 | keratin, type II cytoskeletal 1 [Homo sapiens] | 1358.06 | 61.34% | 1 | 35 | 41 | 66 |  | 644 | 66 | 8.12 |
| 119395754 | keratin, type II cytoskeletal 5 [Homo sapiens] | 307.18 | 31.19% | 16 | 11 | 23 | 27 |  | 590 | 62.3 | 7.74 |
| 119703744 | desmoglein-1 preproprotein [Homo sapiens] | 111.62 | 9.72% | 1 | 8 | 8 | 9 |  | 1049 | 113.7 | 5.03 |
| 119874213 | heat shock 70 kDa protein 12A [Homo sapiens] | 0 | 1.78% | 1 | 1 | 1 | 1 |  | 675 | 74.9 | 6.77 |
| 120431745 | huntingtin-associated protein 1 isoform 4 [Homo sapiens] | 26.98 | 0.67% | 1 | 1 | 1 | 1 |  | 1635 | 183.3 | 5.27 |
| 121114302 | cullin-4B isoform 2 [Homo sapiens] | 58.58 | 10.61% | 2 | 5 | 9 | 9 |  | 895 | 102.2 | 7.94 |
| 122937295 | BEN domain-containing protein 3 [Homo sapiens] | 0 | 1.81% | 1 | 1 | 1 | 1 |  | 828 | 94.4 | 5.43 |
| 123173757 | ribonucleoprotein PTB-binding 1 [Homo sapiens] | 41.81 | 5.69% | 1 | 3 | 3 | 3 |  | 756 | 79.5 | 8.92 |
| 124248516 | neutrophil defensin 1 precursor [Homo sapiens] | 0 | 9.57% | 2 | 1 | 1 | 1 |  | 94 | 10.2 | 6.99 |
| 124487399 | melanoregulin [Homo sapiens] | 0 | 2.42% | 1 | 3 | 3 | 3 |  | 1321 | 150 | 6.83 |
| 124494240 | unconventional myosin-Ic isoform c [Homo sapiens] | 0 | 2.43% | 3 | 3 | 3 | 3 |  | 1028 | 117.8 | 9.41 |
| 125988409 | protein Red [Homo sapiens] | 36.01 | 8.26% | 1 | 5 | 5 | 5 |  | 557 | 65.6 | 6.64 |
| 127139033 | NADPH--cytochrome P450 reductase [Homo sapiens] | 36.1 | 3.24% | 1 | 2 | 2 | 2 |  | 680 | 77 | 5.58 |
| 132566680 | scavenger receptor class B member 1 isoform 2 [Homo sapiens] | 28.14 | 3.16% | 2 | 2 | 2 | 2 |  | 506 | 56.1 | 7.05 |
| 134133220 | protein FAM86B1 [Homo sapiens] | 0 | 6.08% | 5 | 1 | 1 | 1 |  | 296 | 32.8 | 6.44 |
| 148491070 | CTP synthase 1 [Homo sapiens] | 131.18 | 11.84% | 2 | 6 | 6 | 6 |  | 591 | 66.6 | 6.46 |
| 148612853 | ATP-binding cassette sub-family F member 3 [Homo sapiens] | 37.93 | 2.68% | 1 | 2 | 2 | 2 |  | 709 | 79.7 | 6.34 |
| 148727247 | ubiquitin carboxyl-terminal hydrolase 5 isoform 2 [Homo sapiens] | 62.4 | 5.87% | 2 | 4 | 4 | 4 |  | 835 | 93.2 | 5.08 |
| 150417996 | ubiquitin-like modifier-activating enzyme 6 [Homo sapiens] | 0 | 0.86% | 1 | 1 | 1 | 1 |  | 1052 | 117.9 | 6.14 |
| 153070260 | myristoylated alanine-rich C-kinase substrate [Homo sapiens] | 118.37 | 19.58% | 1 | 4 | 4 | 4 |  | 332 | 31.5 | 4.45 |
| 153791300 | protein SDA1 homolog [Homo sapiens] | 46.61 | 1.31% | 1 | 1 | 1 | 1 |  | 687 | 79.8 | 9.25 |
| 153945790 | myosin-8 [Homo sapiens] | 81.03 | 4.85% | 6 | 1 | 8 | 8 |  | 1937 | 222.6 | 5.74 |
| 154146191 | heat shock protein HSP 90-alpha isoform 2 [Homo sapiens] | 658.38 | 32.51% | 3 | 12 | 24 | 34 |  | 732 | 84.6 | 5.02 |
| 154354966 | mitochondrial inner membrane protein isoform 3 [Homo sapiens] | 133.2 | 24.23% | 3 | 18 | 18 | 18 |  | 747 | 82.6 | 6.57 |
| 154355000 | far upstream element-binding protein 2 [Homo sapiens] | 307.26 | 29.54% | 3 | 17 | 17 | 20 |  | 711 | 73.1 | 7.3 |
| 154448890 | THO complex subunit 1 [Homo sapiens] | 0 | 1.98% | 1 | 1 | 1 | 1 |  | 657 | 75.6 | 4.98 |
| 155030185 | protein asunder homolog [Homo sapiens] | 0 | 3.54% | 1 | 2 | 2 | 2 |  | 706 | 80.2 | 6.7 |
| 155030196 | rap1 GTPase-GDP dissociation stimulator 1 isoform 3 [Homo sapiens] | 1265.65 | 16.53% | 4 | 26 | 38 | 67 |  | 2504 | 276.2 | 6.47 |
| 155969697 | keratin, type II cytoskeletal 6C [Homo sapiens] | 345.05 | 30.14% | 18 | 8 | 21 | 23 |  | 564 | 60 | 8 |
| 155969707 | insulin-degrading enzyme isoform 1 [Homo sapiens] | 0 | 1.18% | 1 | 1 | 1 | 1 |  | 1019 | 117.9 | 6.61 |
| 156071459 | ADP/ATP translocase 2 [Homo sapiens] | 35.34 | 6.71% | 4 | 2 | 2 | 2 |  | 298 | 32.8 | 9.69 |
| 156071462 | ADP/ATP translocase 3 [Homo sapiens] | 0 | 3.02% | 2 | 1 | 1 | 1 |  | 298 | 32.8 | 9.74 |
| 156104874 | envoplakin [Homo sapiens] | 0 | 0.49% | 1 | 1 | 1 | 1 |  | 2033 | 231.5 | 6.96 |
| 156105689 | methylmalonyl-CoA mutase, mitochondrial precursor [Homo sapiens] | 0 | 1.87% | 1 | 1 | 1 | 1 |  | 750 | 83.1 | 6.93 |
| 156523968 | poly [ADP-ribose] polymerase 1 [Homo sapiens] | 48.79 | 2.27% | 1 | 2 | 2 | 2 |  | 1014 | 113 | 8.88 |
| 156564401 | vesicle-fusing ATPase [Homo sapiens] | 100.37 | 12.90% | 1 | 9 | 9 | 9 |  | 744 | 82.5 | 6.95 |
| 156616301 | kelch-like protein 32 [Homo sapiens] | 0 | 2.90% | 1 | 1 | 1 | 1 |  | 620 | 70.3 | 6.42 |
| 157388904 | HEAT repeat-containing protein 2 [Homo sapiens] | 0 | 1.05% | 1 | 1 | 1 | 1 |  | 855 | 93.5 | 6.42 |
| 157389005 | calpain-2 catalytic subunit isoform 1 [Homo sapiens] | 46.81 | 6.00% | 2 | 5 | 5 | 5 |  | 700 | 80 | 4.98 |
| 157412270 | heterogeneous nuclear ribonucleoprotein M isoform b [Homo sapiens] | 293.55 | 29.67% | 2 | 18 | 18 | 21 |  | 691 | 73.6 | 8.82 |
| 157738639 | tensin-4 precursor [Homo sapiens] | 0 | 2.24% | 1 | 2 | 2 | 2 |  | 715 | 76.7 | 7.34 |
| 158937236 | puromycin-sensitive aminopeptidase [Homo sapiens] | 121.85 | 12.19% | 1 | 10 | 10 | 10 |  | 919 | 103.2 | 5.72 |
| 161702986 | ezrin [Homo sapiens] | 404.52 | 44.03% | 2 | 16 | 27 | 32 |  | 586 | 69.4 | 6.27 |
| 162951877 | adseverin isoform 1 [Homo sapiens] | 311.64 | 32.87% | 2 | 19 | 19 | 21 |  | 715 | 80.4 | 5.71 |
| 164698500 | septin-9 isoform e [Homo sapiens] | 82.31 | 8.06% | 6 | 3 | 3 | 3 |  | 422 | 47.5 | 6.48 |
| 165905591 | LIM domain and actin-binding protein 1 isoform 3 [Homo sapiens] | 25.62 | 5.00% | 5 | 3 | 3 | 3 |  | 600 | 67.1 | 5.8 |
| 166706909 | interferon-induced protein 44-like [Homo sapiens] | 0 | 2.43% | 1 | 1 | 1 | 1 |  | 452 | 51.3 | 6.79 |
| 166795250 | kinesin-like protein KIF2C [Homo sapiens] | 0 | 1.10% | 1 | 1 | 1 | 1 |  | 725 | 81.3 | 7.83 |
| 167001643 | mevalonate kinase [Homo sapiens] | 43.56 | 4.13% | 1 | 5 | 5 | 5 |  | 1379 | 153.1 | 6.19 |
| 167234419 | thyroid hormone receptor-associated protein 3 [Homo sapiens] | 0 | 0.94% | 1 | 1 | 1 | 1 |  | 955 | 108.6 | 10.15 |
| 167466173 | heat shock 70 kDa protein 1A/1B [Homo sapiens] | 155.81 | 12.95% | 1 | 4 | 7 | 9 |  | 641 | 70 | 5.66 |
| 169646441 | rab GDP dissociation inhibitor beta isoform 2 [Homo sapiens] | 0 | 2.75% | 2 | 1 | 1 | 1 |  | 400 | 45.6 | 6.24 |
| 169790833 | lysosome-associated membrane glycoprotein 2 isoform C precursor [Homo sapiens] | 69.47 | 4.80% | 3 | 2 | 3 | 3 |  | 666 | 74.5 | 5.29 |
| 169790958 | methionine synthase reductase isoform 1 [Homo sapiens] | 129.95 | 15.33% | 2 | 7 | 7 | 7 |  | 698 | 77.6 | 6.49 |
| 170295797 | carbamoyl-phosphate synthase [ammonia], mitochondrial isoform c [Homo sapiens] | 0 | 1.05% | 3 | 1 | 1 | 1 |  | 1049 | 116 | 5.96 |
| 171460918 | dynamin-1-like protein isoform 3 [Homo sapiens] | 234.41 | 25.75% | 7 | 15 | 15 | 16 |  | 699 | 78.1 | 6.81 |
| 178557739 | complement C4-B preproprotein [Homo sapiens] | 1487.14 | 31.59% | 2 | 2 | 43 | 66 |  | 1744 | 192.6 | 7.27 |
| 186910302 | rac GTPase-activating protein 1 [Homo sapiens] | 0 | 2.53% | 1 | 1 | 1 | 1 |  | 632 | 71 | 8.88 |
| 187960067 | metabotropic glutamate receptor 8 isoform a precursor [Homo sapiens] | 32.18 | 0.57% | 2 | 1 | 1 | 1 |  | 1568 | 173.1 | 7.71 |
| 188219762 | denticleless protein homolog [Homo sapiens] | 0 | 1.51% | 1 | 1 | 1 | 1 |  | 730 | 79.4 | 8.87 |
| 188497750 | hexokinase-1 isoform HKI-td [Homo sapiens] | 58.11 | 7.51% | 6 | 6 | 6 | 7 |  | 905 | 101 | 6.9 |
| 188528628 | polyribonucleotide nucleotidyltransferase 1, mitochondrial precursor [Homo sapiens] | 89.08 | 12.26% | 1 | 9 | 9 | 9 |  | 783 | 85.9 | 7.77 |
| 188528667 | histone H3.3C [Homo sapiens] | 41.67 | 5.19% | 5 | 1 | 1 | 1 |  | 135 | 15.2 | 11.11 |
| 188536065 | platelet glycoprotein 4 [Homo sapiens] | 78.61 | 5.93% | 1 | 4 | 4 | 5 |  | 472 | 53 | 7.96 |
| 189083778 | gelsolin isoform b [Homo sapiens] | 299.53 | 19.56% | 6 | 11 | 11 | 11 |  | 731 | 80.6 | 5.85 |
| 189163528 | alpha-1-antitrypsin precursor [Homo sapiens] | 885.41 | 28.80% | 1 | 28 | 28 | 59 |  | 1118 | 117.7 | 5.36 |
| 189458817 | transferrin receptor protein 1 [Homo sapiens] | 170.72 | 22.11% | 1 | 14 | 14 | 14 |  | 760 | 84.8 | 6.61 |
| 189458819 | transferrin receptor protein 1 [Homo sapiens] | 119.75 | 8.16% | 1 | 5 | 5 | 5 |  | 760 | 84.8 | 6.61 |
| 189458821 | protein-glutamine gamma-glutamyltransferase E [Homo sapiens] | 31.99 | 1.01% | 1 | 1 | 1 | 1 |  | 693 | 76.6 | 5.86 |
| 189491778 | nephrocystin-1 isoform 4 [Homo sapiens] | 0 | 1.14% | 4 | 1 | 1 | 1 |  | 614 | 69.9 | 5.07 |
| 190014586 | PC4 and SFRS1-interacting protein isoform 1 [Homo sapiens] | 31 | 2.70% | 2 | 1 | 1 | 1 |  | 333 | 37.7 | 9.17 |
| 190341074 | pyridoxal-dependent decarboxylase domain-containing protein 1 [Homo sapiens] | 33.12 | 3.17% | 9 | 2 | 2 | 2 |  | 788 | 86.7 | 5.38 |
| 190358504 | SHC SH2 domain-binding protein 1 [Homo sapiens] | 0 | 1.19% | 1 | 1 | 1 | 1 |  | 672 | 75.6 | 4.75 |
| 192447426 | disks large homolog 4 isoform 2 [Homo sapiens] | 31.25 | 1.11% | 7 | 1 | 1 | 1 |  | 721 | 80.1 | 5.94 |
| 192448443 | peptidyl-prolyl cis-trans isomerase FKBP10 precursor [Homo sapiens] | 64.67 | 4.81% | 1 | 3 | 3 | 3 |  | 582 | 64.2 | 5.62 |
| 193083131 | MAP/microtubule affinity-regulating kinase 3 isoform e [Homo sapiens] | 516.04 | 23.93% | 4 | 25 | 32 | 40 |  | 1258 | 139.4 | 9.25 |
| 193794814 | fructose-bisphosphate aldolase A isoform 1 [Homo sapiens] | 23.29 | 3.85% | 2 | 1 | 1 | 1 |  | 364 | 39.4 | 8.09 |
| 194018511 | keratin, type II cytoskeletal 1b [Homo sapiens] | 234.51 | 8.30% | 4 | 2 | 7 | 10 |  | 578 | 61.9 | 5.99 |
| 194097352 | alpha-actinin-1 isoform c [Homo sapiens] | 145.42 | 22.55% | 5 | 4 | 17 | 20 |  | 887 | 102.6 | 5.5 |
| 194097354 | eukaryotic peptide chain release factor GTP-binding subunit ERF3A isoform 2 [Homo sapiens] | 32.23 | 2.99% | 4 | 2 | 2 | 2 |  | 636 | 68.5 | 5.33 |
| 194440727 | dynein heavy chain 12, axonemal isoform 1 [Homo sapiens] | 0 | 0.26% | 1 | 1 | 1 | 1 |  | 3092 | 356.7 | 6.19 |
| 195972866 | keratin, type I cytoskeletal 10 [Homo sapiens] | 967.83 | 50.34% | 15 | 27 | 31 | 57 |  | 584 | 58.8 | 5.21 |
| 201861823 | fermitin family homolog 2 isoform 3 [Homo sapiens] | 0 | 1.58% | 3 | 1 | 1 | 1 |  | 633 | 72.4 | 7.5 |
| 205277386 | glutamine--fructose-6-phosphate aminotransferase [isomerizing] 1 isoform 2 [Homo sapiens] | 175.34 | 13.95% | 3 | 7 | 7 | 8 |  | 681 | 76.7 | 6.84 |
| 205277463 | transketolase isoform 1 [Homo sapiens] | 158.57 | 21.19% | 2 | 11 | 11 | 11 |  | 623 | 67.8 | 7.66 |
| 205360954 | polycystin-1 isoform 1 precursor [Homo sapiens] | 456.73 | 4.86% | 8 | 8 | 21 | 32 |  | 4853 | 522.9 | 6.52 |
| 208431836 | polyadenylate-binding protein 4 isoform 3 [Homo sapiens] | 316.17 | 34.71% | 3 | 11 | 19 | 21 |  | 631 | 69.5 | 9.52 |
| 208973244 | 14-3-3 protein zeta/delta [Homo sapiens] | 44.65 | 16.73% | 7 | 3 | 3 | 3 |  | 245 | 27.7 | 4.79 |
| 209413730 | poliovirus receptor isoform gamma precursor [Homo sapiens] | 53.15 | 3.85% | 3 | 1 | 1 | 1 |  | 364 | 39.3 | 6.21 |
| 209862831 | annexin A2 isoform 2 [Homo sapiens] | 43.65 | 14.75% | 2 | 4 | 4 | 4 |  | 339 | 38.6 | 7.75 |
| 209869993 | trifunctional purine biosynthetic protein adenosine-3 isoform 1 [Homo sapiens] | 32.96 | 3.27% | 4 | 3 | 3 | 3 |  | 1010 | 107.7 | 6.7 |
| 209969703 | protein RCC2 [Homo sapiens] | 0 | 1.34% | 1 | 1 | 1 | 1 |  | 522 | 56 | 8.78 |
| 209969812 | KN motif and ankyrin repeat domain-containing protein 2 isoform 2 [Homo sapiens] | 0 | 1.41% | 2 | 1 | 1 | 1 |  | 851 | 91.1 | 5.63 |
| 212549561 | ataxin-1-like [Homo sapiens] | 0 | 1.89% | 1 | 1 | 1 | 1 |  | 689 | 73.3 | 6.6 |
| 214830187 | spartin [Homo sapiens] | 30.96 | 3.60% | 1 | 2 | 2 | 2 |  | 666 | 72.8 | 5.91 |
| 217035118 | TBC1 domain family member 23 isoform 2 [Homo sapiens] | 23.77 | 2.05% | 2 | 1 | 1 | 1 |  | 684 | 76.5 | 5.4 |
| 218749883 | endoplasmic reticulum mannosyl-oligosaccharide 1,2-alpha-mannosidase [Homo sapiens] | 31.49 | 1.29% | 1 | 1 | 1 | 1 |  | 699 | 79.5 | 7.72 |
| 221316614 | extracellular matrix protein 1 isoform 1 precursor [Homo sapiens] | 0 | 1.19% | 1 | 1 | 1 | 1 |  | 758 | 85.4 | 6.46 |
| 221316616 | extracellular matrix protein 1 isoform 2 precursor [Homo sapiens] | 0 | 3.13% | 3 | 1 | 1 | 1 |  | 415 | 46.1 | 6.39 |
| 221625538 | MORC family CW-type zinc finger protein 1 [Homo sapiens] | 0 | 1.12% | 1 | 1 | 1 | 1 |  | 984 | 112.8 | 7.85 |
| 223029424 | large subunit GTPase 1 homolog [Homo sapiens] | 40.15 | 2.89% | 1 | 2 | 2 | 2 |  | 658 | 75.2 | 6.38 |
| 223029519 | conserved oligomeric Golgi complex subunit 2 isoform 2 [Homo sapiens] | 0 | 1.76% | 2 | 1 | 1 | 1 |  | 737 | 83.1 | 6.62 |
| 223278387 | calmodulin-like protein 5 [Homo sapiens] | 0 | 9.59% | 1 | 1 | 1 | 1 |  | 146 | 15.9 | 4.44 |
| 223555917 | protein LYRIC [Homo sapiens] | 61.31 | 6.53% | 1 | 3 | 3 | 3 |  | 582 | 63.8 | 9.32 |
| 223718019 | rod cGMP-specific 3',5'-cyclic phosphodiesterase subunit beta isoform 3 [Homo sapiens] | 0 | 1.39% | 3 | 1 | 1 | 1 |  | 575 | 66.5 | 5.41 |
| 223972653 | aldehyde dehydrogenase family 16 member A1 isoform 2 [Homo sapiens] | 0 | 3.86% | 2 | 3 | 3 | 3 |  | 751 | 79.9 | 7.58 |
| 225735593 | RNA polymerase II-associated protein 3 isoform 2 [Homo sapiens] | 74.19 | 6.50% | 3 | 3 | 3 | 3 |  | 631 | 71.8 | 7.64 |
| 225903424 | putative deoxyribonuclease TATDN2 [Homo sapiens] | 0 | 3.15% | 1 | 1 | 1 | 1 |  | 761 | 85 | 7.39 |
| 225903430 | mannosyl-oligosaccharide glucosidase isoform 2 [Homo sapiens] | 64.05 | 1.64% | 2 | 1 | 1 | 1 |  | 731 | 80.7 | 7.11 |
| 226056130 | prostaglandin reductase 1 isoform 2 [Homo sapiens] | 45.02 | 3.32% | 2 | 1 | 1 | 1 |  | 301 | 32.9 | 7.2 |
| 226246608 | putative RNA polymerase II subunit B1 CTD phosphatase RPAP2 [Homo sapiens] | 0 | 2.29% | 1 | 1 | 1 | 1 |  | 612 | 69.5 | 7.78 |
| 228008396 | heterogeneous nuclear ribonucleoprotein Q isoform 2 [Homo sapiens] | 61.62 | 9.48% | 7 | 2 | 4 | 4 |  | 464 | 52 | 9.16 |
| 229577436 | spermatogenesis-associated protein 5-like protein 1 [Homo sapiens] | 26.23 | 1.06% | 1 | 1 | 1 | 1 |  | 753 | 80.7 | 8.09 |
| 238859647 | probable G-protein coupled receptor 128 precursor [Homo sapiens] | 0 | 1.51% | 1 | 1 | 1 | 1 |  | 797 | 88.9 | 8.46 |
| 239757335 | PREDICTED: uncharacterized protein C19orf68 [Homo sapiens] | 0 | 1.73% | 1 | 1 | 1 | 1 |  | 578 | 64.3 | 8.87 |
| 239937451 | adenosylhomocysteinase isoform 2 [Homo sapiens] | 0 | 2.23% | 2 | 1 | 1 | 1 |  | 404 | 44.6 | 6.47 |
| 242247001 | clathrin heavy chain 2 isoform 2 [Homo sapiens] | 29.13 | 0.76% | 3 | 1 | 1 | 1 |  | 1583 | 180.2 | 5.78 |
| 253970504 | RNA-binding protein EWS isoform 4 [Homo sapiens] | 153.64 | 11.33% | 5 | 5 | 5 | 7 |  | 600 | 62.5 | 9.39 |
| 256419001 | double-stranded RNA-binding protein Staufen homolog 2 isoform d [Homo sapiens] | 0 | 2.01% | 9 | 1 | 1 | 3 |  | 398 | 43.3 | 9.42 |
| 256818782 | PAP-associated domain-containing protein 5 isoform b [Homo sapiens] | 32.37 | 1.38% | 2 | 1 | 1 | 1 |  | 651 | 70.8 | 9.51 |
| 260436846 | zinc finger and SCAN domain-containing protein 30 [Homo sapiens] | 293.89 | 4.25% | 1 | 2 | 5 | 12 |  | 1152 | 127.5 | 6.42 |
| 260436922 | suprabasin isoform 1 precursor [Homo sapiens] | 29.25 | 7.08% | 1 | 2 | 2 | 2 |  | 1271 | 138.2 | 7.34 |
| 260898763 | RNA-binding protein 28 isoform 2 [Homo sapiens] | 0 | 1.13% | 2 | 1 | 1 | 1 |  | 618 | 69.9 | 8.54 |
| 262527235 | lysosomal protective protein isoform c precursor [Homo sapiens] | 0 | 1.07% | 2 | 1 | 1 | 1 |  | 1408 | 158.1 | 5.55 |
| 262527293 | ras-related protein Rab-35 isoform 2 [Homo sapiens] | 24.6 | 7.24% | 30 | 1 | 1 | 1 |  | 152 | 17 | 6.79 |
| 264681410 | inner nuclear membrane protein Man1 isoform 2 [Homo sapiens] | 0 | 1.32% | 1 | 1 | 1 | 1 |  | 910 | 99.8 | 7.78 |
| 264681565 | xaa-Pro aminopeptidase 1 isoform 2 [Homo sapiens] | 41.54 | 7.79% | 2 | 4 | 4 | 4 |  | 642 | 72.1 | 6.02 |
| 268840316 | interleukin-1 receptor accessory protein isoform 1 precursor [Homo sapiens] | 0 | 1.58% | 1 | 1 | 1 | 3 |  | 570 | 65.4 | 8.12 |
| 283046706 | la-related protein 4 isoform d [Homo sapiens] | 0 | 3.95% | 6 | 2 | 2 | 2 |  | 582 | 64.5 | 6 |
| 283046708 | la-related protein 4 isoform e [Homo sapiens] | 0 | 2.92% | 6 | 1 | 1 | 1 |  | 445 | 49.9 | 5.43 |
| 283135173 | RNA-binding protein FUS isoform 3 [Homo sapiens] | 42.75 | 2.68% | 5 | 1 | 1 | 1 |  | 522 | 53.2 | 9.36 |
| 283806701 | zinc finger BED domain-containing protein 1 [Homo sapiens] | 25.66 | 1.30% | 1 | 1 | 1 | 1 |  | 694 | 78.1 | 6.1 |
| 283806705 | spermatid perinuclear RNA-binding protein isoform 2 [Homo sapiens] | 29.02 | 5.17% | 2 | 1 | 3 | 3 |  | 658 | 71.9 | 8.65 |
| 285002233 | glycerol-3-phosphate dehydrogenase, mitochondrial precursor [Homo sapiens] | 53.4 | 4.40% | 1 | 3 | 3 | 3 |  | 727 | 80.8 | 7.69 |
| 288541299 | protein NOXP20 [Homo sapiens] | 54.88 | 8.17% | 1 | 4 | 4 | 4 |  | 563 | 60.7 | 4.68 |
| 289176994 | phosphorylase b kinase regulatory subunit alpha, skeletal muscle isoform isoform 3 [Homo sapiens] | 25.87 | 0.61% | 4 | 1 | 1 | 1 |  | 1151 | 129.3 | 5.96 |
| 289577114 | eukaryotic translation initiation factor 4 gamma 2 isoform 2 [Homo sapiens] | 38.4 | 2.42% | 2 | 2 | 2 | 2 |  | 869 | 98.1 | 6.99 |
| 289629267 | protein transport protein Sec23B isoform 2 [Homo sapiens] | 0 | 1.47% | 2 | 1 | 1 | 1 |  | 749 | 84.4 | 6.99 |
| 294997308 | dystroglycan preproprotein [Homo sapiens] | 0 | 2.12% | 1 | 2 | 2 | 2 |  | 895 | 97.5 | 8.56 |
| 295986608 | immunoglobulin lambda-like polypeptide 5 isoform 1 [Homo sapiens] | 170.86 | 26.64% | 2 | 4 | 4 | 6 |  | 214 | 23 | 8.84 |
| 297374791 | fragile X mental retardation protein 1 isoform ISO9 [Homo sapiens] | 41.06 | 1.88% | 3 | 1 | 1 | 1 |  | 586 | 66.2 | 8.47 |
| 299758423 | E3 ubiquitin-protein ligase LRSAM1 isoform 2 [Homo sapiens] | 39.92 | 3.30% | 2 | 2 | 2 | 2 |  | 696 | 80.4 | 6 |
| 300192933 | AFG3-like protein 2 [Homo sapiens] | 103.58 | 11.04% | 4 | 9 | 9 | 9 |  | 797 | 88.5 | 8.66 |
| 301171475 | ATP-dependent RNA helicase DDX3X isoform 3 [Homo sapiens] | 201.69 | 17.18% | 8 | 9 | 10 | 11 |  | 646 | 71.3 | 6.62 |
| 301336155 | tRNA (cytosine(34)-C(5))-methyltransferase isoform 2 [Homo sapiens] | 0 | 6.69% | 2 | 4 | 4 | 4 |  | 732 | 82.3 | 7.01 |
| 307078125 | clathrin interactor 1 isoform 3 [Homo sapiens] | 51.92 | 3.04% | 3 | 2 | 2 | 2 |  | 625 | 68.1 | 6.42 |
| 308044526 | ankyrin repeat and KH domain-containing protein 1 isoform 4 [Homo sapiens] | 0 | 1.20% | 5 | 1 | 1 | 1 |  | 581 | 59.7 | 4.48 |
| 308193325 | trypsin-3 isoform 4 preproprotein [Homo sapiens] | 77.76 | 5.42% | 4 | 1 | 1 | 1 |  | 240 | 25.9 | 5.83 |
| 308199423 | glutamate--cysteine ligase catalytic subunit isoform b [Homo sapiens] | 51.06 | 6.01% | 2 | 3 | 3 | 3 |  | 599 | 68.6 | 5.94 |
| 308737003 | origin recognition complex subunit 3 isoform 3 [Homo sapiens] | 32.31 | 1.41% | 3 | 1 | 1 | 1 |  | 568 | 65.9 | 7.62 |
| 310750368 | ensconsin isoform 8 [Homo sapiens] | 0 | 1.82% | 9 | 1 | 1 | 1 |  | 603 | 66.9 | 9.51 |
| 310923189 | leptin receptor isoform 4 precursor [Homo sapiens] | 1084.94 | 16.36% | 13 | 28 | 34 | 66 |  | 1993 | 220.2 | 6.61 |
| 311893345 | copine-1 isoform c [Homo sapiens] | 0 | 1.68% | 3 | 1 | 1 | 1 |  | 536 | 58.9 | 5.83 |
| 311893365 | calpain-1 catalytic subunit [Homo sapiens] | 138.55 | 15.13% | 1 | 9 | 9 | 9 |  | 714 | 81.8 | 5.67 |
| 315113878 | receptor-type tyrosine-protein phosphatase-like N isoform 2 precursor [Homo sapiens] | 2088.08 | 24.75% | 4 | 44 | 50 | 120 |  | 1673 | 178 | 7.15 |
| 316659409 | actin, cytoplasmic 2 [Homo sapiens] | 154.53 | 25.33% | 14 | 7 | 7 | 10 |  | 375 | 41.8 | 5.48 |
| 316983158 | NADH-ubiquinone oxidoreductase 75 kDa subunit, mitochondrial isoform 3 [Homo sapiens] | 73.69 | 5.84% | 5 | 3 | 3 | 3 |  | 616 | 67.5 | 5.33 |
| 320461711 | peroxiredoxin-1 [Homo sapiens] | 28.77 | 8.04% | 2 | 2 | 2 | 2 |  | 199 | 22.1 | 8.13 |
| 321400140 | CD44 antigen isoform 7 precursor [Homo sapiens] | 119.63 | 10.00% | 6 | 3 | 3 | 4 |  | 340 | 37.3 | 5.52 |
| 323714253 | lysosome membrane protein 2 isoform 2 precursor [Homo sapiens] | 47.09 | 7.16% | 2 | 4 | 4 | 4 |  | 335 | 37.7 | 5.14 |
| 324120938 | disks large homolog 1 isoform 5 [Homo sapiens] | 51.08 | 1.52% | 5 | 1 | 1 | 1 |  | 788 | 87.2 | 6.55 |
| 325197164 | eukaryotic initiation factor 4A-I isoform 2 [Homo sapiens] | 0 | 4.90% | 2 | 1 | 1 | 1 |  | 347 | 39.5 | 5.82 |
| 334191701 | 6-phosphofructokinase type C isoform 2 [Homo sapiens] | 41.88 | 2.45% | 5 | 2 | 2 | 2 |  | 776 | 85.3 | 8.59 |
| 336176066 | RNA-binding protein 39 isoform d [Homo sapiens] | 31.06 | 7.57% | 4 | 3 | 3 | 3 |  | 502 | 56.3 | 10.04 |
| 338968934 | cold shock domain-containing protein E1 isoform 2 [Homo sapiens] | 44.22 | 7.95% | 4 | 6 | 6 | 6 |  | 767 | 85.7 | 6.2 |
| 341913933 | PREDICTED: hypothetical protein LOC100653084 [Homo sapiens] | 73.39 | 7.89% | 2 | 1 | 2 | 6 |  | 279 | 31.5 | 7.78 |
| 341913935 | PREDICTED: putative V-set and immunoglobulin domain-containing protein 7 [Homo sapiens] | 0 | 9.17% | 2 | 1 | 1 | 1 |  | 120 | 13.4 | 8.18 |
| 341913937 | PREDICTED: putative V-set and immunoglobulin domain-containing protein 6-like [Homo sapiens] | 0 | 8.47% | 1 | 1 | 1 | 1 |  | 189 | 20.9 | 9.35 |
| 341914409 | PREDICTED: ig kappa chain V-III region VG-like [Homo sapiens] | 51.44 | 5.92% | 1 | 1 | 1 | 1 |  | 152 | 16.6 | 7.08 |
| 341914862 | PREDICTED: hypothetical protein LOC100291917 [Homo sapiens] | 96.43 | 11.19% | 3 | 2 | 4 | 6 |  | 295 | 33.3 | 9.22 |
| 341914865 | PREDICTED: ig heavy chain V-III region VH26-like [Homo sapiens] | 57.42 | 12.72% | 1 | 1 | 2 | 3 |  | 173 | 19.1 | 9.82 |
| 341914877 | PREDICTED: immunoglobulin omega chain-like [Homo sapiens] | 26.52 | 9.52% | 1 | 2 | 2 | 2 |  | 147 | 16.4 | 8.16 |
| 341914924 | PREDICTED: ig heavy chain V-III region VH26-like [Homo sapiens] | 73.92 | 11.83% | 3 | 1 | 2 | 8 |  | 186 | 20.3 | 9.03 |
| 341914926 | PREDICTED: ig heavy chain V-III region VH26-like [Homo sapiens] | 0 | 4.76% | 1 | 1 | 1 | 1 |  | 231 | 25.3 | 7.15 |
| 341914938 | PREDICTED: putative HERC2-like protein 3-like [Homo sapiens] | 0 | 3.28% | 2 | 1 | 1 | 2 |  | 244 | 27.9 | 8.53 |
| 341914941 | PREDICTED: ig heavy chain V-III region VH26-like [Homo sapiens] | 67.28 | 17.69% | 4 | 1 | 3 | 4 |  | 147 | 16.1 | 9.28 |
| 341915156 | PREDICTED: ig kappa chain V-III region VH-like [Homo sapiens] | 29.99 | 5.96% | 3 | 1 | 1 | 2 |  | 151 | 16.6 | 7.12 |
| 341915168 | PREDICTED: ig kappa chain V-I region Walker-like [Homo sapiens] | 32.53 | 13.01% | 5 | 1 | 1 | 1 |  | 123 | 13.3 | 8.31 |
| 341915780 | PREDICTED: ig heavy chain V-III region VH26-like [Homo sapiens] | 67.62 | 14.86% | 1 | 1 | 2 | 3 |  | 148 | 16.3 | 8.37 |
| 341916194 | PREDICTED: complement C4-A isoform 1 [Homo sapiens] | 180.13 | 9.00% | 4 | 1 | 12 | 13 |  | 1744 | 192.7 | 7.08 |
| 343403796 | NEDD8 ultimate buster 1 isoform 2 [Homo sapiens] | 36.74 | 1.28% | 2 | 1 | 1 | 1 |  | 625 | 71.9 | 6.62 |
| 347543829 | dihydropyrimidinase-related protein 2 isoform 3 [Homo sapiens] | 0 | 2.99% | 7 | 1 | 1 | 1 |  | 536 | 58.1 | 6.15 |
| 349585060 | disabled homolog 2 isoform 2 [Homo sapiens] | 0 | 1.74% | 2 | 1 | 1 | 1 |  | 749 | 80.2 | 5.9 |
| 351721595 | tripartite motif-containing protein 3 isoform 2 [Homo sapiens] | 0 | 1.60% | 2 | 1 | 1 | 1 |  | 625 | 67.7 | 8.63 |
| 354983501 | protein-L-isoaspartate(D-aspartate) O-methyltransferase isoform 1 [Homo sapiens] | 0 | 2.99% | 2 | 3 | 3 | 3 |  | 1138 | 129.2 | 6.79 |
| 357588516 | colipase isoform 3 preproprotein [Homo sapiens] | 0 | 0.89% | 3 | 1 | 1 | 1 |  | 1234 | 137.4 | 6.28 |
| 367460087 | myosin-10 isoform 2 [Homo sapiens] | 54.93 | 1.06% | 3 | 1 | 1 | 1 |  | 1976 | 228.9 | 5.54 |
| 372466577 | keratin, type II cytoskeletal 8 isoform 2 [Homo sapiens] | 111.73 | 8.49% | 15 | 1 | 9 | 9 |  | 483 | 53.7 | 5.59 |
| 375331941 | dipeptidyl peptidase 3 isoform 2 [Homo sapiens] | 148.63 | 21.64% | 2 | 11 | 11 | 11 |  | 707 | 79.3 | 5.03 |
| 375477430 | T-complex protein 1 subunit delta isoform b [Homo sapiens] | 0 | 1.57% | 2 | 1 | 1 | 1 |  | 509 | 54.7 | 7.83 |
| 378744163 | zinc finger protein 195 isoform 6 [Homo sapiens] | 0 | 3.27% | 3 | 4 | 4 | 4 |  | 1376 | 159.3 | 7.64 |
| 378744165 | RNA polymerase II-associated factor 1 homolog isoform 2 [Homo sapiens] | 77.15 | 14.85% | 2 | 5 | 5 | 5 |  | 485 | 55.4 | 5.36 |
| 380714663 | cullin-3 isoform 2 [Homo sapiens] | 52.06 | 1.85% | 3 | 1 | 1 | 1 |  | 702 | 81 | 8.51 |
| 382546190 | ATP synthase subunit alpha, mitochondrial isoform c [Homo sapiens] | 0 | 6.56% | 3 | 3 | 3 | 3 |  | 503 | 54.5 | 8.24 |
| 384871702 | DNA mismatch repair protein Msh2 isoform 2 [Homo sapiens] | 0 | 1.27% | 2 | 1 | 1 | 1 |  | 868 | 97.3 | 5.76 |
| 385198097 | shootin-1 isoform e [Homo sapiens] | 39.34 | 8.03% | 5 | 4 | 4 | 4 |  | 498 | 56.3 | 5.71 |
| 386642860 | threonine--tRNA ligase, cytoplasmic isoform 1 [Homo sapiens] | 426.69 | 37.62% | 3 | 27 | 27 | 32 |  | 723 | 83.4 | 6.67 |
| 391353390 | signal recognition particle subunit SRP72 isoform 2 [Homo sapiens] | 169.47 | 12.62% | 2 | 7 | 7 | 7 |  | 610 | 67.8 | 9.41 |
| 395132436 | 60S ribosomal protein L18 isoform 2 [Homo sapiens] | 30.85 | 8.18% | 2 | 1 | 1 | 1 |  | 159 | 18.1 | 11.84 |
| 409971397 | dnaJ homolog subfamily C member 10 isoform 2 precursor [Homo sapiens] | 46.96 | 2.41% | 2 | 2 | 2 | 2 |  | 747 | 86.1 | 7.39 |
| 410169914 | PREDICTED: uncharacterized protein LOC642131 [Homo sapiens] | 21.51 | 5.46% | 3 | 1 | 1 | 1 |  | 293 | 32.5 | 9.94 |
| 410170898 | PREDICTED: complement C4-A isoform 2 [Homo sapiens] | 1454.72 | 30.86% | 4 | 1 | 42 | 65 |  | 1698 | 187.6 | 7.12 |
| 422398894 | cytoplasmic dynein 1 intermediate chain 2 isoform 4 [Homo sapiens] | 124.47 | 8.35% | 9 | 5 | 5 | 5 |  | 611 | 68.3 | 5.29 |
| 431822408 | heat shock protein HSP 90-beta isoform c [Homo sapiens] | 894.76 | 40.90% | 3 | 16 | 29 | 57 |  | 714 | 82.3 | 5.06 |
| 440309857 | heat shock protein 75 kDa, mitochondrial isoform 2 [Homo sapiens] | 231.16 | 20.58% | 2 | 11 | 12 | 13 |  | 651 | 74.2 | 7.87 |
| 441478305 | glutamine--tRNA ligase isoform b [Homo sapiens] | 145.05 | 15.45% | 2 | 10 | 10 | 10 |  | 764 | 86.5 | 7.05 |
| 503775288 | RING finger protein 214 isoform 2 [Homo sapiens] | 0 | 1.82% | 2 | 1 | 1 | 1 |  | 548 | 61.3 | 8.13 |
| 509155825 | uncharacterized protein C12orf42 isoform 2 [Homo sapiens] | 0 | 4.53% | 2 | 1 | 1 | 1 |  | 265 | 28.6 | 9.55 |
|  |  |  |  |  |  |  |  |  |  |  |  |
